# Do cancer biomarkers make targeted therapies cost-effective? A systematic review in metastatic colorectal cancer

**DOI:** 10.1371/journal.pone.0204496

**Published:** 2018-09-26

**Authors:** Mikyung Kelly Seo, John Cairns

**Affiliations:** 1 Department of Health Services Research and Policy, Faculty of Public Health and Policy, London School of Hygiene and Tropical Medicine, London, United Kingdom; 2 Centre for Cancer Biomarkers, Faculty of Medicine, University of Bergen, Bergen, Norway; University of South Alabama Mitchell Cancer Institute, UNITED STATES

## Abstract

**Background:**

Recent advances in targeted therapies have raised expectations that the clinical application of biomarkers would improve patient’s health outcomes and potentially save costs. However, the cost-effectiveness of biomarkers remains unclear irrespective of the cost-effectiveness of corresponding therapies. It is thus important to determine whether biomarkers for targeted therapies provide good value for money. This study systematically reviews economic evaluations of biomarkers for targeted therapies in metastatic colorectal cancer (mCRC) and assesses the cost-effectiveness of predictive biomarkers in mCRC.

**Methods:**

A literature search was performed using Medline, Embase, EconLit, NHSEED. Papers published from 2000 until June 2018 were searched. All economic evaluations assessing biomarker-guided therapies with companion diagnostics in mCRC were searched. To make studies more comparable, cost-effectiveness results were synthesized as per biomarker tests and corresponding therapies. Methodological quality was assessed using the Quality of Health Economic Studies (QHES) instrument.

**Results:**

Forty-six studies were included in this review. Of these, 17 studies evaluated the intrinsic value of cancer biomarkers, whereas the remaining studies focused on assessing the cost-effectiveness of corresponding drugs. Most studies indicated favourable cost-effectiveness of biomarkers for targeted therapies in mCRC. Some studies reported that biomarkers were cost-effective, while their corresponding therapies were not cost-effective. A considerable number of economic evaluations were conducted in pre-defined genetic populations and thus, often failed to fully capture the biomarker’s clinical and economic values. The average QHES score was 73.6.

**Conclusion:**

Cancer biomarkers for targeted therapies in mCRC were mostly found to be cost-effective; otherwise, they at least improved the cost-effectiveness of targeted therapies by saving some costs. However, this did not necessarily make their corresponding therapies cost-effective. While companion biomarkers reduced therapy costs, the savings were not sufficient to make the corresponding agents cost-effective. Evaluation of biomarkers was often restricted to the cost of tests and did not consider their clinical values or biomarker prevalence.

## Introduction

Colorectal cancer (CRC) is the third most common cancer and the third leading cause of cancer deaths worldwide [1]. In Europe, it is the most common cause of cancer death after lung cancer. In 2012, 241,600 men and 205,200 women were diagnosed with CRC [2], and 113,200 men and 101,500 women died from CRC [2]. In the USA, 136,830 cases newly diagnosed with CRC and 50,310 deaths with CRC were projected in 2014 [3].

Despite recent developments in targeted therapies, gene sequencing and molecular diagnostics, promising optimized and personalized treatment regimens tailored for individual patients, CRC remains one of the less treatable cancers. Most cases of CRC are sporadic and develop slowly over several years, progressing through a series of clinical and histopathological stages from single crypt lesions through benign adenomas to malignant carcinomas, as a result of an accumulation of mutations in tumor suppressor genes and oncogenes or a genetic instability [4, 5]. The 5-year survival rate for early-stage CRC is about 90% but it falls to 10% for late-stage CRC metastasized to distant sites [6] and cancer mortality is mainly due to metastasis [7, 8].

There are multiple treatments available for patients with metastatic colorectal cancer (mCRC), including targeted therapies guided by biomarkers [9–11]. Recent advances in targeted therapies have raised expectations that clinical application of biomarkers might improve health benefits while avoiding unnecessary toxicity and adverse events. It can potentially reduce health care system costs by containing unnecessary costs without hurting patient health outcomes [12].

These therapies comprise epidermal growth factor receptor (EGFR), vascular endothelial growth factor (VEGF), and tyrosine kinase (TK) inhibitors. VEGF-targeted therapies include bevacizumab, aflibercept, and ramucirumab. EGFR inhibitors are cetuximab and panitumumab. Regorafenib is a TK inhibitor. Of these, only anti-EGFR therapies have a predictive biomarker clearly established for guiding treatment options as an integral part of the clinical pathways [13, 14]. Current guidelines in Europe and the USA recommend that all mCRC patients receive Kirsten rat sarcoma (KRAS) testing prior to treatment with EGFR inhibitors since KRAS mutation status–wild type (WT) or mutant (MT)–predicts the response to anti-EFGR therapies [15, 16]. Recently, the testing was expanded to RAS testing (both KRAS and NRAS) [17]. KRAS and NRAS mutations serve as predictive biomarkers for anti-EGFR therapies, only patients with RAS wild-type tumors benefit from these therapies. No positive predictive biomarkers exist yet, that identify eligible patients rather than exclude ineligible patients. No other molecular marker is part of routine clinical practice when deciding optimized and tailored treatment regimens for mCRC patients. However, irinotecan is a biomarker-directed chemotherapy for treating mCRC, which unlike molecularly targeted therapies, is a cytotoxic drug given to get rid of or control cancer cells. UGT1A1 testing showed clinical benefits for the administration of irinotecan [18]. All these predictive biomarkers are currently used in clinical settings to make treatment decisions for the safe and effective use of targeted therapies in treating mCRC.

Third-party payers often prioritize competing interventions by assessing cost-effectiveness using cost-effectiveness (CEA) and cost-utility analysis (CUA) [19]. The former is often assessed per additional life-years gained (LYs), and the latter per additional quality-adjusted life-year (QALY). Incremental differences in costs and benefits between alternative interventions are the main focus of economic evaluations and thus, the primary study outcome is usually to estimate the incremental cost-effectiveness ratio (ICER) per LYs or QALYs [19]. The comparison of alternative courses of action for cancer biomarkers for targeted therapies can be broadly categorised into two forms: ‘test-treat’ strategy (patients are treated with new intervention guided by biomarker status) and ‘treat-all’ strategy (all patients are treated without biomarker testing) [20].

To sum up, the use of biomarkers may permit optimising regimens without compromising health outcomes. This has significant implications for healthcare payers in containing expenditures that provide no or minimal benefits to patients. Despite such high expectations, the cost-effectiveness of cancer biomarkers remains unclear given that they are often co-assessed as part of high cost targeted therapy. This study systematically reviews economic evaluations of biomarker-guided therapies and aims to determine the impact of companion biomarkers on the cost-effectiveness of the corresponding therapies in mCRC.

## Materials and methods

### Literature search

A systematic literature search on the cost-effectiveness of cancer biomarkers for targeted therapies in mCRC was performed using Medline (Ovid), Embase (Ovid), EconLit, and the National Health Service Economic Evaluation Database (NHSEED) in June 2018. The search terms (S2 Table) were validated by an information specialist. The reference lists of relevant articles were scrutinized, and the grey literature was hand-searched.

The electronic search was performed using Medical subject heading (MeSH) terms and keywords that were developed based on patients (mCRC), intervention (cancer biomarkers for targeted therapies), and outcome (ICERs). These were combined with free-word texts using relevant economic terms (e.g. “cost-effectiveness”) and the drug names of targeted therapies both in brand and generic terms. Targeted therapies granted a marketing authorization with companion biomarkers by the European Medicines Agency (EMA) or US Food and Drug Administration (FDA) were included in the literature search strategy [13]. Studies published in English were searched from 2000 until June 2018.

### Study selection

The study selection was based on the inclusion and exclusion criteria formulated by the PICOS framework i.e., population, intervention, comparator, outcome, study type (S3 Table). Given the companion nature of predictive biomarkers for targeted therapies, their cost-effectiveness is interconnected with clinical effectiveness and costs of corresponding therapies as well as biomarker tests. Hence, the cost-effectiveness of biomarker testing as well as corresponding agents were included in this review. Selection of papers followed the eligibility criteria below:

1. Population: the intervention is being applied to adult patients with a diagnosis of mCRC.
2. Intervention: cancer biomarkers for targeted therapies (predictive/companion biomarkers). These biomarkers are used as diagnostic tools to guide treatment or select patients responsive to subsequent corresponding therapies. Cancer biomarkers without market authorizations co-licensed with targeted therapies were excluded.
3. Comparator: conventional treatments or targeted therapies with or without use of biomarker tests.
4. Outcome: ICERs for LYs, ICERs for QALYs. Studies merely reporting costs or effectiveness were excluded.
5. Study type: economic evaluations including model or trial-based analyses. Studies merely reporting on methodological issues, reviews, comments, letters or editorials were excluded.

The study selection had three main stages. Firstly, search hits from the electronic databases were imported into EndNote and duplicate citations were removed. Secondly, the titles and abstracts of the identified articles were screened independently by two reviewers. Studies clearly indicated as irrelevant were excluded. Thirdly, the full articles retrieved that met the inclusion criteria were screened by two reviewers, with any disagreements between reviewers resolved by discussion.

### Data extraction

A data extraction form was created based on the Cochrane Handbook of Systematic Reviews of Interventions and the CHEERS statement [21, 22]. The following items were extracted: publication details, target patients, interventions, comparators, outcomes (ICERs), study designs. Data extraction was performed by the first assessor (MKS) using Microsoft Excel® and any ambiguities were resolved by discussion with the second reviewer (JC).

### Quality assessment

The methodological quality of the included studies was assessed using the Quality of Health Economic Studies (QHES) scales [23]. The QHES has been validated and shown to be useful in discriminating higher quality economic evaluation studies from poorer ones [24]. The quality assessment was conducted by two assessors (MKS, JC). Since no standardized interpretation of QHES scores exist, we assigned QHES scores to three quality groups; above 70 scores as high quality, between 50 and 70 as fair quality, and below 50 as poor quality studies. Final QHES score per study was resolved by discussion.

### Synthesizing data

The cost-effectiveness results of included studies divided into two groups: 1) the cost-effectiveness of cancer biomarkers for targeted therapies (predictive/companion biomarkers), 2) the cost-effectiveness of targeted therapies licensed with companion biomarkers. ICERs for companion biomarkers are the primary outcome of this study and those for targeted therapies are a secondary outcome.

To enhance the comparability of heterogeneous cost-effectiveness studies especially for the primary outcome of this review, the cost-effectiveness results for companion biomarkers were qualitatively synthesized by the strategies compared in economic evaluations as described below.

- ‘Test-treat’ strategy: Biomarker test performed, and therapy guided by the biomarker results; for example, RAS wild-type patients receive new intervention (i.e. targeted therapies) and RAS mutant patients receive standard care (i.e. existing therapies/best supportive care (BSC)/chemotherapy)
- ‘Treat-all’ with new therapy strategy: No biomarker test performed, and all patients treated with new intervention
- ‘Treat-all’ with standard care strategy: No biomarker test performed, all patients treated with standard care

## Results

### Literature search and study selection

**Fig 1:**
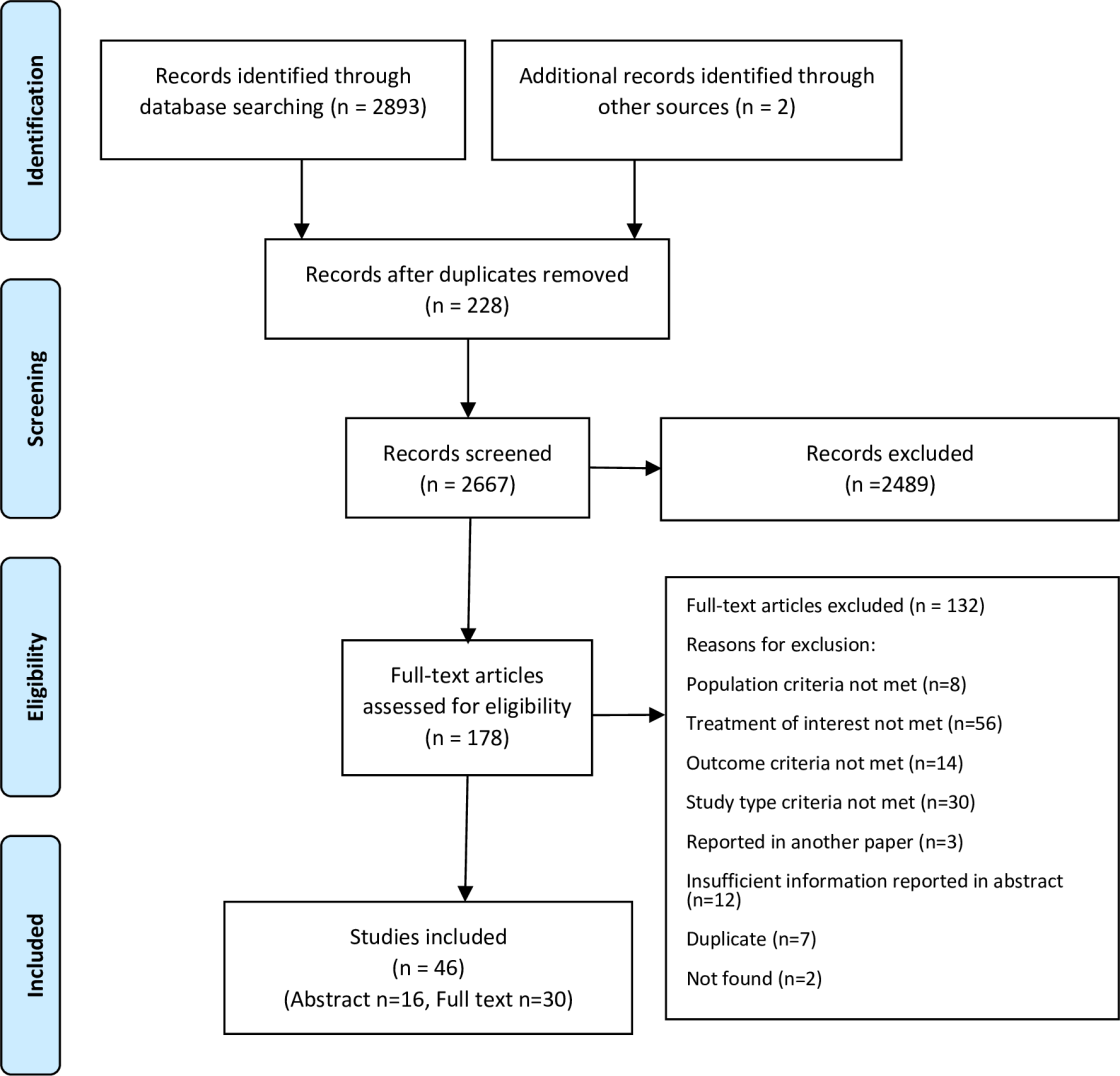
PRISMA flow diagram of study selection.

The electronic search located 2893 publications, and reference tracking identified two additional articles. Duplicates (228 papers) were removed, resulting in 2667 unique studies. The titles and abstracts were then assessed according to the pre-determined eligibility criteria, and 2489 papers were excluded. A total of 178 papers were selected for full-text assessment. Main reasons for exclusion were the type of intervention studied (i.e. not related to cancer biomarkers for targeted therapies) and the study type (i.e. not economic evaluations or cost-effectiveness analyses). Fifteen papers were excluded because the results were reported in another paper or insufficient information was reported in abstract only. Fourteen papers were excluded as they did not report ICERs as their study outcome. Eight papers were additionally excluded because they did not target patients with mCRC. Altogether, 46 publications were included in the review, consisting of 30 studies reported in full text and 16 reported in abstract only. Study selection is presented in a PRISMA flow diagram (Fig 1)

### Overview of included studies

**Fig 2:**
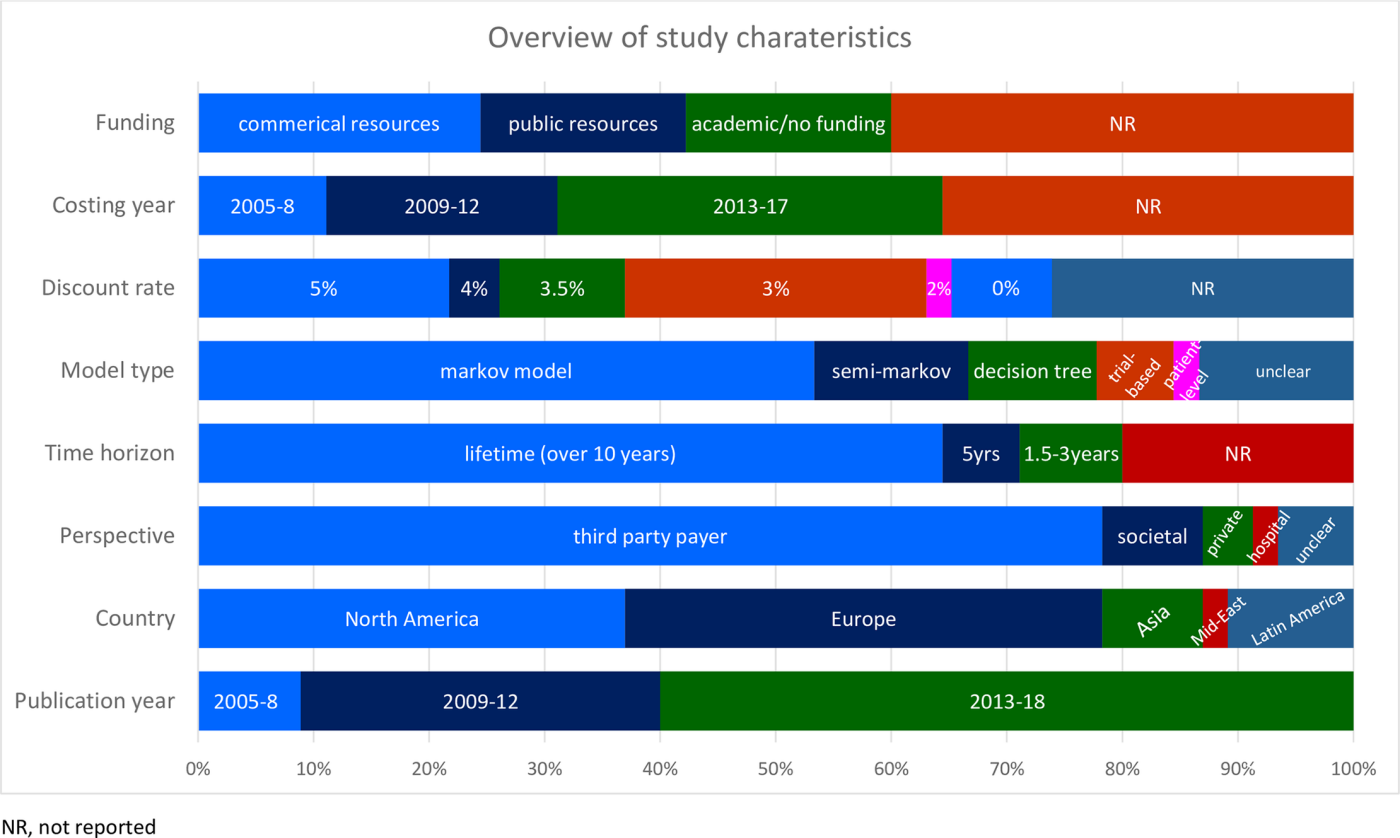
Overview of study characteristics.

The modelling designs, the intervention strategies, and the comparator strategies of the included cost-effectiveness studies were heterogeneous. The majority of studies were model-based economic evaluations except for three trial-based studies. Analyses involved comparisons between two and seven strategy arms. Most studies employed the perspective of third party payers (79%), while only a small proportion of studies adopted a societal perspective (8%) and patient or hospital perspectives. The type of perspective was not disclosed in three studies [25–27]. Most of the included studies were modelled for lifetime or more than 10-year time horizons (66%), while trial-based analyses were modelled only for their trial periods, i.e. 1.5 or 2 years. Most of the studies were set in Europe (40%) and North America (35%), except for six in Latin America, five in Asia, and one in the Middle East. Manufacturer sponsorship was declared by 13 studies, while most studies were either funded by public or academic resources (nine studies from public resources, eight studies from either academic resources or no external funding). Most abstracts did not declare funding source for their projects. Moreover, three full papers did not declare their source of funding. Study characteristics are synthesized in Fig 2 and detailed characteristics for each study are provided in S4 Table. No economic evaluations of cancer biomarkers for targeted therapies in mCRC were published before 2005. Many studies were published in recent years, 60% after 2012. Four studies appeared between 2005–08, 14 studies in 2009–12, and 28 studies in 2013–18. Likewise, recent years were used in costing years of assessments; the years of 2005–08 in five studies, 2009–12 in nine studies, and 2013–18 in sixteen studies. However, a considerable number of assessments did not specify a base year for pricing (17 studies).

### Primary synthesis

#### Cost-effectiveness of predictive biomarkers in mCRC

Seventeen studies investigated the cost-effectiveness of cancer biomarkers for targeted therapies (Tables 1–3) (detailed results of ICERs per study are provided in S5 Table). These studies assessed the cost-effectiveness of predictive (companion) biomarkers aside from that of the corresponding therapies. Overall, all studies showed favourable results toward predictive biomarkers. Thirteen studies found biomarker testing to be cost-effective [28–40], of which four studies reported biomarker testing to be dominant [33–36]. Five studies showed cost-saving [41–45] compared to that of ‘no-testing’. Wen et al. [32] evaluated cost-effectiveness of RAS screening prior to monoclonal antibodies and found that RAS testing before cetuximab is more cost-effective compared to KRAS-testing with cetuximab. After re-calculating their ICERs, we concluded that all strategies they used were well beyond the acceptable willingness to pay thresholds in China, but RAS testing appeared to be more favourable than KRAS testing for patients with mCRC. Some studies reported conflicting results of cost-effectiveness between predictive biomarkers and corresponding therapies; the biomarkers were cost-effective, but their corresponding therapies were not [41–43, 34]. Existing predictive biomarkers (or companion diagnostics) co-licensed with targeted therapies in mCRC included KRAS and RAS approved for the use of panitumumab and cetuximab, and UGT1A1 genotyping approved for the administration of irinotecan. KRAS and RAS testing was the most frequently evaluated in economic evaluations (KRAS testing in eight studies; RAS testing in seven studies) and UGT1A1 testing in four studies.

**Table 1:** Cost-effectiveness finding of KRAS testing for corresponding targeted therapies.

Study	Strategy comparison	Model type, time horizon	ICER/LYs (re-caculated if necessary)	ICER/QALYs (re-caculated if necessary)	Currency,year	Conclusion based on outcome
***‘Test-treat’ strategy compared to ‘treat-all’ patients with standard care without testing***						
Behl et al. 2012 [41]	KRAS testing plus Cmab vs. Treat all with BSC	Markov model, 10-year	672,216*	NA	US$, 2010	The use of KRAS testing was cost-saving prior to Cmab however, Cmab plus KRAS testing was not cost-effective.
Blank et al. 2011 [28]	KRAS testing plus Cmab vs. Treat all with BSC	Markov model, Lifetime	NA	63,647*	Euro, NR	KRAS testing prior to Cmab is clinically appropriate and economically favourable.
Carlson J.J. 2010 [42]	KRAS testing plus Cmab vs. Treat all with BSC	Decision analytic model, NR	NA	264,644	US$, NR	KRAS testing was cost-saving but Cmab plus KRAS testing was not cost-effective.
Health Quality Ontario 2010 [29]	KRAS testing plus Cmab vs. Treat all with BSC	Markov model, Lifetime	NA	54,802	CA$, 2009	KRAS testing was cost-effective for all strategies considered.
	KRAS testing plus Pmab vs. Treat all with BSC		NA	47,795	CA$, 2009	
	KRAS testing plus Cmab + Irinotecan vs. Treat all with BSC		NA	42,710	CA$, 2009	
Shiroiwa et al. 2010 [34]	KRAS testing plus Cmab vs. No-KRAS testing (Treat all with BSC)	Markov model, 2.5- years	120,000	180,000	US$, 2010	KRAS testing strategy was dominant compared to no-KRAS testing strategy. However, Cmab (with or without KRAS testing) was not cost-effective.
***‘Test-treat’ strategy compared to ‘treat-all’ patients with new treatment without testing***						
Niedersuess-Beke D. et al. 2015 [44]	KRAS testing + Pmab or Cmab vs. No predictive biomarker testing (Cmab/Pmab all)	NR, NR	26,276	NA	EU€, 2013	Testing predictive biomarkers is cost-saving.
***‘Treat-all’ patients with new treatment without testing compared to ‘test-treat’ strategy***						
Behl et al. 2012 [41]	Treat all with Cmab vs. KRAS testing plus Cmab	Markov model, 10-years	2,932,767	NA	US$, 2010	Treating all patients with Cmab without testing was not cost-effective; no-testing is not cost-effective.
Blank et al. 2011 [28]	Treat all with Cmab vs. KRAS testing plus Cmab	Markov model, Lifetime	NA	314,588	Euro, NR	Treating all patients with Cmab without testing was not cost-effective.
Health Quality Ontario 2010 [29]	Treat all with Cmab vs. KRAS testing plus Cmab	Markov model, Lifetime	NA	Dominated	CA$, 2009	No-testing was not cost-effective.
	Treat all with Cmab vs. KRAS testing plus Pmab		NA	308,236	CA$, 2009	
	Treat all with Cmab vs. KRAS testing plus Cmab + Irinotecan		NA	163,396	CA$, 2009	
Vijayaraghavan et al. 2012 [35]	Treat all with Cmab/Pmab/Combination therapy vs. KRAS testing plus Cmab/Pmab/Combination therapy	Markov model, Lifetime	Higher costs, same effectiveness	NA	US$, 2009; EU€ 2009	No-testing was not cost-effective (dominated).
***Pre-defined genetic population (KRAS WT patients)***						
Harty et al. 2015 [43]	Cmab + FOLFIRI vs. FOLFIRI; strategies compared between different cohorts of patients stratified by different biomarker status including KRAS WT group	NR, NR	NA	72,053	GB£, NR	Cmab plus chemotherapy was not cost-effective in a subgroup of patients with KRAS WT. However, the stratification of patients by genetic biomarker status does improve the cost-effectiveness of corresponding therapies.

*ICERs were re-calculated using total costs and effects provided in the pertinent paper.

AB; abstract, NA; not available, NR; not reported

**Table 2:** Cost-effectiveness finding of RAS testing for corresponding targeted therapies.

Study	Comparison	Model type, time horizon	ICER/LYs (re-caculated if necessary)	ICER/QALYs (re-caculated if necessary)	Currency, year	Conclusion based on outcome
***‘Test-treat’ strategy compared to ‘treat-all’ patients with standard care without testing***						
Wu et al. [40]	Cmab + FOLFIRI vs. FOLFIRI	Markov model, 10-year	$12,107	$14,049	US$, 2016	RAS testing with Cmab is cost-effective when patient assistance programme is available in China.
***‘Test-treat’ strategy compared to ‘treat-all’ patients with new treatment without testing***						
Niedersuess-Beke D. et al. 2015 [44]	RAS testing + Pmab or Cmab vs. No predictive biomarker testing (Cmab/Pmab all)	NR, NR	9,686	NA	EU€, 2013	Predictive biomarker testing were cost-saving; RAS testing scenario showed lower ICERs than KRAS testing scenario.
Saito et al. 2017 [31]	RAS testing vs. No testing before EGFR therapies	Markov model, 5-year	2,574,111	3,049,132	JP¥, NR	RAS testing was cost-effective compared to no-testing; however, comprehensive profiling is more cost-effective than RAS testing only.
***Pre-defined genetic population (RAS WT patients)***						
Harty et al. 2015 [43]	Cmab + FOLFIRI vs. FOFIRI for patients stratified into RAS WT group	NR, NR	NA	44,184	GB£, NR	Stratification of patients by genetic biomarker status improved cost-effectiveness of Cmab; however, its ICERs was yet beyond the £20,000-£30,000 thresholds for UK. Recently however, NICE committees accepted that it was a life-extending end-of-life treatment and approved under the exceptional thresholds of £50,000 in UK[47].
Souza et al. 2017 [39]	Cmab + Chemotherapy vs. Chemotherapy alone	Markov model, 20-year	NA	56,750	BRL$, NR	The addition of Cmab to the standard chemotherapy is a cost-effective therapy for RAS WT patients with liver-limited disease.
Wen et al. 2015 [32]	RAS-Cmab vs.KRAS-Cmab	Markov model, 10-years	NA	17710*	US$, 2014	Patients treated with Cmab and RAS-testing was more cost-effective against the strategy of KRAS-testing and treated with Cmab.
	RAS-Bmab vs. KRAS-Bmab		NA	71079*		Patients with RAS-testing and treated with Bmab was not cost-effective compared to KRAS testing and treated with Bmab.
Zhou et al. 2016 [36]	RAS-Cmab vs.KRAS-Cmab	Markov model, Lifetime	NA	(22450)*	US$, NR (2016 assumed)	RAS screening was dominant over KRAS testing.
	RAS-Bmab vs. KRAS-Bmab		NA	(3966)*		

*ICERs were re-calculated using total costs and effects provided in the pertinent paper.

AB; abstract, NA; not available, NR; not reported

**Table 3:** Cost-effectiveness of UGT1A1 testing.

Study	Comparison	Model type, time horizon	ICER/LYs (re-calculated if necessary)	ICER/QALYs (re-calculated if necessary)	Currency, year	Conclusion based on outcome
***‘Test-treat’ strategy versus ‘treat all’ patients with standard care without testing***						
Butzke 2016 [33]	UGT1A1 genotyping and dose reduction vs. the current standard of no testing	Markov model, Lifetime	NA	Dominant	EU€, 2013	UGT1A1 testing dominates the strategy of no-testing strategy in treating patients with irinotecan-based chemotherapy.
Gold et al. 2009 [37]	UGT1A1 testing and dose reduction of irinotecan vs. the current standard of no testing	Decision-analytic model, 5-year	NA	Favorable	US$, 2007	UGT1A1 testing could be cost-effective if irinotecan dose reduction does not reduce efficacy.
Obradovic et al. 2008 [45]	UGT1A1 testing and dose reduction of irinotecan vs. No UGT1A1 testing and standard care of irinotecan	Decision analytic model, Lifetime	Cost-saving (African, Caucasian)	NA	US$, 2006	Genotyping with dose reduction of irinotecan was cost-saving for the population of African/Caucasian however, not cost-effective for Asian populations.
			6,818,203 (Asian)	NA		
Pichereau et al. 2010 [30]	UGT1A1 genotyping before irinotecan vs. no genotyping strategy	Decision tree, Lifetime	942.8–1090.1	NA	EU€, 2006	Genotyping strategy was cost-effective compared to no-testing strategy.

NA; not available or not applicable

#### Cost-effectiveness of KRAS testing

All studies reported favourable cost-effectiveness for KRAS testing prior to the administration of the corresponding targeted therapies, while four corresponding therapies were not cost-effective (Table 1). KRAS testing for targeted therapies was assessed mostly to pre-select eligible patients before administering EGFR therapies such as cetuximab or panitumumab. As shown in Table 1, all studies suggested favourable cost-effectiveness for the use of KRAS testing in administering EGFR therapies. Although 50% of these studies reported the corresponding targeted therapies as not cost-effective [41–43, 34], they found that KRAS testing was cost-effective (n = 4) or at least cost-saving (n = 4) prior to the provision of corresponding therapies.

Although all studies suggested favourable cost-effectiveness of KRAS testing before providing EGFR therapies, the inclusion of KRAS biomarker testing did not necessarily ensure the cost-effectiveness of the costly corresponding targeted therapies. For example, Behl et al. [41] evaluated the cost-effectiveness of KRAS testing to select patients responsive to cetuximab compared to administering cetuximab to all patients without testing. We re-calculated their ICERs in order to evaluate cost-effectiveness using an appropriate strategy comparison such as ‘test-treat’ strategy against ‘treating all patients with BSC without testing’ strategy. KRAS testing plus administering cetuximab had a lower ICER ($672,216) than treating all patients with cetuximab with no KRAS testing ($827,913), when both strategies were compared against the reference strategy of not providing cetuximab at all. It confirms that KRAS testing saved some costs by restricting cetuximab to particular patients, however cetuximab is yet far beyond the acceptable cost-effectiveness thresholds of USA.

Carlson[42] compared two intervention strategies (1. Cetuximab for all patients, 2. Cetuximab for KRAS wild-type and BSC for KRAS mutant patients based on biomarker testing) compared to BSC for all patients without biomarker testing. Neither intervention strategy was cost-effective. However, the KRAS testing strategy saved $10,037 with a negligible decrease in QALYs compared to the cetuximab for all patients strategy. Likewise, Shiroiwa and colleagues [34] conducted a comparative analysis using the same strategies; 1) KRAS-testing strategy, 2) No KRAS-testing strategy (cetuximab for all), 3) No cetuximab strategy (BSC for all). They found the KRAS-testing strategy dominated the no-KRAS-testing (cetuximab for all) strategy, however, the ICER for cetuximab (with or without KRAS testing) was too high even if treatments were limited to KRAS wild-type patients. Meanwhile, Harty and colleagues [43] investigated the cost-effectiveness of cetuximab in combination with irinotecan when patients were stratified into different genetic biomarker groups and suggested that the use of a biomarker improved the cost-effectiveness of cetuximab but its ICER was beyond acceptable thresholds for UK.

To sum up, targeted therapies were never cost-effective when a ‘no-testing strategy (treating all patients with new therapy)’ was compared to a ‘test-treat’ strategy. This confirms that KRAS testing is a better use of resources than ‘no-testing’ prior to the administration of targeted therapies. However, when a ‘test-treat’ strategy was compared to ‘treat all with BSC/SOC’, there were conflicting results; three studies not cost-effective [41, 42, 34] and two studies favourable [28, 29]. This implies a positive impact of KRAS testing in improving the cost-effectiveness of its companion therapies however; it does not necessarily mean that KRAS testing can ensure the cost-effectiveness of subsequent targeted therapy.

#### Cost-effectiveness of RAS testing

Seven studies evaluated the cost-effectiveness of RAS testing and most of them found favourable results for RAS biomarker testing (Table 2). Of these, two studies assessed the cost-effectiveness of RAS screening compared with that of KRAS testing with targeted therapies [32, 36]. Both studies were performed from a Chinese health care system perspective and found that RAS testing was cost-effective compared to KRAS testing with cetuximab. However, Wu et al. [40] found that RAS testing with cetuximab is only cost-effective when a patient assistance programme is available in China. However, Wen et al. [32] found that bevacizumab with RAS testing was not cost-effective compared to bevacizumab with KRAS testing. They reported $74,600 which is far more than three times Chinese GDP per capita ($24,000 [46]).

However, most of these studies did not use an appropriate strategy comparison such as evaluating a ‘test-treat’ strategy in comparison to a ‘treat all with existing standard therapy’. Two studies were compared against ‘treat all with new therapy’, and four studies were performed in a pre-defined genetic population. Only one recent study employed a comparative strategy of chemotherapy alone without mutation testing [40], however, this economic evaluation was of relatively low quality. Thus, the evidence on cost-effectiveness of RAS testing is still inconclusive. Further evaluation is required using an appropriate comparator strategy of ‘treat all patients with standard care without testing’ instead of ‘treating all with new therapy without testing’.

#### Cost-effectiveness of UGT1A1 testing

The four studies assessing UGT1A1 genotyping for the administration of irinotecan found that the genotyping was either cost-saving or cost-effective (Table 3). However, Obradovic et al. [45] reported that UGT1A1 genotyping in combination with a reduced dose of irinotecan was not cost-effective for Asian population groups, reporting very high ICERs at $6,818,000. Since all studies were conducted for populations in Europe or USA, further research on Asian populations to confirm this difference in cost-effectiveness of UGT1A1 testing may be required before deciding to reduce irinotecan doses.

All studies compared alternative strategies correctly, between ‘test-treat’ with new intervention and ‘treat all’ patients with standard care without testing. For example, Gold and colleagues [37] assessed the comparative analysis of UGT1A1 testing and no testing prior to irinotecan administration, using different scenarios of dose reduction efficacy of irinotecan. They reported that, assuming no reduction in treatment efficacy, the average cost savings of the genotyping test were $272.34 with 0.073 quality-adjusted days saved. Most recently, Butzke et al. [33] evaluated the UGT1A1 genotyping from a German statutory health insurance perspective and found that genotyping prior to irinotecan-based chemotherapy dominates non-guided colon cancer care in Germany. However, this study also reported that there is substantial structural uncertainty in relation to the degree of dose-reduction in heterozygotic patients and suggested to validate it in clinical practice whether physicians indeed chose to reduce dosing in both heterozygote and homozygote patients.

Overall, UGT1A1 testing appears to be cost-effective prior to the administration of irinotecan, especially in relation to dose reduction and prevention of adverse events. However, two studies used narrow health outcome measures such as neutropenia avoided [45, 30] and one study suggested a conditional cost-effectiveness of UGT1A1 testing depending on the treatment efficacy of irinotecan dose reduction.

### Secondary synthesis

#### Cost-effectiveness of targeted therapies licensed with companion biomarkers

In 29 studies, the cost-effectiveness of targeted therapies was evaluated [48–56, 25, 57–60, 26, 27]. This secondary synthesis analyses economic evaluations of targeted therapies which did not explicitly analyse the value of predictive biomarkers as part of assessing the cost-effectiveness of biomarker-guided therapies. Fifty-nine percent of these economic evaluations reported favourable cost-effectiveness findings for targeted therapies licensed with companion biomarkers in treating mCRC (n = 17). 41% reported that targeted therapies were not cost-effective (n = 12).

76% of these studies (n = 22) performed their comparative analyses in a pre-defined genetic population such as biomarker-positive patients and often, no differences in the value of predictive biomarkers were modelled. These studies frequently assumed that the study population (in all strategy arms) was tested before entering the economic models. However, all studies related to UGT1A1 testing considered the intrinsic value of UGT1A1 testing as an integral part of their comparative analysis in administering irinotecan-based chemotherapies. Among the remaining seven studies, treatment decisions in four studies [48, 61, 62, 25] depended on biomarker mutational status, but in three studies [63–65] the comparative strategies employed were not clear.

Overall, this secondary synthesis found that the inclusion of predictive biomarkers improved the cost-effectiveness of targeted therapies, but the improvement was insufficient to make the corresponding targeted therapies cost-effective. It may imply that the impact of their high drug costs on the cost-effectiveness of targeted therapies is much greater than that of the health benefits gained from pre-selection of responsive patients guided by biomarkers.

Table 4 presents the cost-effectiveness results for targeted therapies labelled with predictive biomarkers (the ICERs are reported in S5 Table). In the case of bevacizumab, which has not yet an established biomarker in clinical settings, it was often assessed as a comparator strategy (n = 8) and not often as an intervention strategy. But two studies compared all three therapies (cetuximab, panitumumab and bevacizumab) and found bevacizumab to be cost-effective [66, 67]. Both studies were conducted in a pre-defined group of patients with KRAS wild-type status. All 29 studies included either cetuximab or panitumumab in their comparative assessments.

**Table 4:** Cost-effectiveness of targeted therapies licensed with companion biomarkers.

Study (reference)	Treatments/Strategies	Model type, time horizon	Biomarker test	Outcome measure	Conclusion based on outcome
Annemans et al. 2007 [48]	1. Cmab + Irinotecan (6 week rule, 12 week rule) 2. Current treatment	Trial-based model, NR	NS	LYs	Cmab + Irinotecan is cost-effective in Belgium.
Asseburg et al. 2011 [49]	1. Cmab + FOLIFIRI 2. Bmab + FOLFOX	Patient-level simulation, 10-year	KRAS	LYs	First line treatment with Cmab plus FOLFIRI offers a cost-effective treatment option versus Bmab plus FOLFOX for KRAS WT genotype patients in Germany. Thus, KRAS testing should be performed on all presenting cases of mCRC to ensure access to this treatment option.
Carvalho et al. 2017 [68]	1. Pmab 2. Cmab 3. BSC	Markov model, Lifetime	RAS	LYs	Both Pmab and Cmab are not cost-effective in patients with RAS WT mCRC.
Chaugule et al. 2012 [69]	1. Cmab + BSC 2. BSC alone	Markov model, Lifetime	KRAS	QALYs	Cmab is not cost-effective in KRAS WT patients with mCRC.
Davari et al. 2015 [63]	1. FOLFIRI, FOLFOX, CAPOX without the addition of Cmab 2. FOLFIRI, FOLFOX, CAPOX with the addition of Cmab	Unclear, NR	KRAS	LYs, QALYs	Addition of Cmab to FOLFIRI, FOLFOX, CAPOX (Capecitabin+oxaliplati) is not cost effective.
Dos Santos et al. 2015 [50]	1. Pmab + mFOLFOX6 2. Bmab + mFOLFOX6	Markov model, Lifetime	RAS	LYs, QALYs	Pmab is clearly cost-effective compared to Bmab for treatment of wild-type RAS mCRC in Brazil.
Ewara et al. 2014 [66]	1. Bmab + FOLFIRI 2. Cmab + FOLFIRI 3. Pmab + FOLFIRI	Markov model, Lifetime	KRAS	QALYs	Bmab+FOLFIRI is cost-effective. Bmab + FOLFIRI found to be dominant over the other two strategies. The other two strategies are dominated by Bmab + FOLFIRI. However, sensivitiy analysis showed that Cmab + FOLIFIRI is being cost-effective under certain range of parameter values—thus, further investigation needed for Cmab.
Graham et al. 2014 [51]	1. Pmab 2. Bmab	Semi-Markov model, Lifetime	KRAS, RAS	LYs, QALYs	Pmab plus mFOLFOX represents good value for money compared to a current SOC Bmab plus mFOLFOX6.
Graham et al. 2016 [52]	1. Panitumumab in pts with KRAS WT status 2. Cetuximab in pts with KRAS WT status	Semi-Markov model, Lifetime	KRAS	LYs, QALYs	Compared to Cmab, the study suggested that Pmab is favorable.
Hnoosh et al. 2015 (AWMSG) [53]	1. Cmab + either FOLFOX, FOLFIRI, CAPOX 2. FOLFOX 3. FOLFIRI 4. CAPOX	Markov model, 10-year	RAS	QALYs	Cmab is cost-effective and a good use of NHS Wales resource through stratifiation of RAS WT patients.
Hnoosh et al. 2015 (NICE) [70]	1. Cmab + either FOLFOX, FOLFIRI, CAPOX 2. FOLFOX 3. FOLFIRI	Markov model, 10-year	RAS	QALYs	Cost-effectiveness of Cmab could be deemed favourable when considering it as end-of-life medicine.
Hoyle et al. 2013 [61]	1. Cmab 2. Cmab + Irinotecan 3. Pmab 4. BSC	Semi-Markov model, 10 years (lifetime)	KRAS	LYs, QALYs	All three strategies (Cmab, Cmab+Irinotecan, Pmab) are not cost-effective.
Huxley et al. 2017 [62]	1. FOLFOX (reference strategy) 2. Cmab + FOLFOX 3. Pmab + FOLFOX	Semi-Markov model, 30 years (lifetime)	RAS	QALYs	Cmab and Pmab in combination with chemotherapy are likely to be poor value for money.
Junqueira et al. 2015 (RAS subgroup) [54]	1. Cmab + FOLIFIRI 2. FOLFIRI	Markov model, 10 years	RAS	LYs	Cmab+FOLIFIRI is cost-effective for a subgroup of patients with RAS wild-type.
Junqueira et al. 2015 (Cmab and Bmab) [55]	1.Cmab+FOLFIRI 2.Bmab+FOLFIRI	Markov model, 10 years	RAS	LYs	The use of Cmab shown significant and meaningful benefits while being cost-saving to HCS in Brazil.
Kourlaba et al. 2014 [56]	1. Pmab + FOLFOX6 2. Bmab + FOLFOX6	Markov model, NR	RAS	QALYs	Pmab + mFOLFOX6 is cost-effective.
Krol et al. 2015 [71]	1. Cmab + FOLFIRI 2. FOLFIRI 3. Cmab + FOLFOX 4. FOLFOX	Markov model, 20-year	RAS	QALYs	ICUR results were close to CET. ICURs strongly differed from the Netherlands and Belgium. It is mainly due to lower drug costs in Belgium.
Lawrence et al. 2013 [67]	1. FBC (reference) 2. Bmab + FBC 3. Cmab + FBC 4. Pmab + FBC	Markov model, Lifetime (to maximum of 10 years)	KRAS	QALYs	Bmab + FBC offers the best value for money in KRAS WT patient population.
Mittmann 2009 [72]	1. Cmab + BSC 2. BSC	Trial-based model, Duration of the clinical trial (18–19 months)	KRAS	LYs, QALYs	ICER of Cmab over BSC alone for unselected mCRC pts was high and sensitive to drug costs. ICER was lower when the analysis was limited to pts with KRAS WT.
Moreno et al. 2012 [25]	1. Scenario A: KRAS WT pts receive weekly Cmab + FOLFOX 2. Scenario B. Pmab + FOLFOX 3. Scenario C. Cmab biweekly + FOLFOX	Unclear, NR	KRAS	Response rate	1st line oxaplatin combinations of biweekly Cmab for WT and Bmab for MT optimise cost per additional response rate rather than Pmab-based schedules.
Norum J. 2006 [64]	1. 3rd line chemotherapy (Cmab + Irinotecan) 2. No 3rd line chemotherapy	Decision tree, Unclear	EGFR	LYs	Cmab + Irinotecan as 3rd line therapy in mCRC is promising, but a very expensive antibody. Reduced drug cost and/or improved overall survival may alter this conclusion.
Ortendahl et al. 2014 [26]	1. FOLFIRI + Cmab 2. FOLFIRI + Bmab	Unclear, Lifetime	KRAS, RAS	LYs, QALYs	Cmab + FOLFIRI improve health outcomes and use financial resource more efficiently compared to Bmab + FOLFIRI.
Riesco-Martinez 2016 [73]	Strategy 1 (reference strategy: EGFRI monotherapy in 3rd line). Strategy 2 (EGFRI and Irinotecan in 3L). Strategy 3 (EGFRI in 1L).	Markov model, 5-year	KRAS, RAS	QALYs	1st line of EGFRI is not cost-effective at its current pricing relative to Bmab.
Rivera et al. 2017 [57]	1. Pmab + mFOLFOX6 2. Bmab + mFOLFOX6	Semi-Markov model, Lifetime	RAS	LYs, QALYs	Pmab+mFOLFOX6 is more cost-effective than Bmab+mFOLFOX6 for the first line treatment of RAS WT mCRC.
Samyshkin et al. 2011 [58]	1. Bmab + Chemotherapy 2. Cmab + Chemotherapy 3. Pmab + Chemotherapy	semi-Markov model, Lifetime	KRAS	QALYs	Cmab plus FOLFIRI is the most cost-effective for patients with KRAS WT tumors. ICERs of Cmab + Chemotherapy (CT), Bmab + CT, and Pmab + CT are within the commonly accepted threshold of CE in UK.
Shankaran et al. 2015 [59]	1. FOLFIRI plus Cmab in treatment-naïve patients with KRAS wt type in mCRC 2. FOLFIRI plus Bmab treatment-naïve patients with KRAS wt type in mCRC	Decision tree, 2 years (trial period)	KRAS, RAS	LYs, QALYs	Results were more favorable for Cmab in RAS-WT patients.
Starling et al. 2007 [65]	1. Cmab + Irinotecan 2. Active/best supportive care (ASC/BSC)	Trial-based model, Lifetime	EGFR	LYs, QALYs	ICERs for Cmab+Irinotecan is relatively high compared to other healthcare interventions.
Vargas-Valencia et al. 2015 [27]	1. Pmab + FOLFOX 2. Cmab + FOLFIRI	Markov model, Lifetime	RAS	LYs	Pmab showed treatment outcomes improvement vs. Cmab for RAS WT patients at a lower cost per life year.
Xu et al. 2016 [60]	1. Pmab 2. Cmab	Markov model, 3-year	NR	LYs, QALYs	Pmab dominates over Cmab. Pmab has a cost advantage over Cmab.

AB; abstract, ASC/BSC; active/best supportive care, Bmab; bevacizumab, Cmab; cetuximab, Pmab; panitumumab and NR; not reported

Cetuximab was assessed in the most studies (n = 24). More studies found cetuximab not to be cost-effective (14 versus 10 studies finding it cost-effective). Among the studies reporting cetuximab as cost-effective, seven studies (78%) were conducted in a pre-defined genetic population either KRAS wild-type or RAS wild-type, and two not [48, 25]. Moreno and colleagues [25] evaluated weekly and biweekly administration of cetuximab compared to panitumumab, where patients in both arms receive biomarker-guided therapies (either cetuximab or panitumumab) when KRAS wild-type and receive bevacizumab when KRAS mutant. They found that biweekly cetuximab for KRAS wild-type and bevacizumab for patients with KRAS mutant status more cost-effective compared to panitumumab-based schedules. Annemans et al. [48] assessed the cost-effectiveness of cetuximab in combination with irinotecan-based chemotherapy compared to current care in Belgium and found that the cetuximab strategy is cost-effective with ICERs between €17000 (6-week treatment scenario) and €40000 (12-week treatment scenario) per LY gained. In this study, all patients in the intervention arm were treated with cetuximab plus irinotecan-based chemotherapy, while patients in the comparator arm were all treated with the current treatment. Nevertheless, none of these studies considered the clinical utility of predictive biomarkers in guiding the optimization of treatments depending on biomarker status in patients.

Among fourteen studies reporting cetuximab as not cost-effective, ten studies were in a pre-defined genetic group and often, this population scoping was used to justify not considering the intrinsic value of predictive biomarkers in the evaluation. Only two studies made the appropriate comparison of a ‘test-treat’ strategy and a ‘treat all with standard of care’. Both were conducted from a perspective of the English NHS and both found cetuximab not cost-effective [61, 62]. Hoyle et al. [61] assessed the cost-effectiveness of cetuximab, cetuximab plus irinotecan, and panitumumab for KRAS wild-type patients from the perspective of the English National Health Service (NHS) and found that all three strategies were not cost-effective compared to BSC. They modelled that 54% of patients were KRAS wild-type and thus, costing £296 per person for KRAS testing (£160 per test). Most recently, Huxley et al. [62] evaluated cetuximab and panitumumab for patients with RAS wild-type mCRC, using a similar comparison structure with Hoyle et al., and they also found that cetuximab and panitumumab in combination of chemotherapy were poor value for money in the English NHS.

Panitumumab assessed in 14 studies, was found to be cost-effective in eight studies [50–52, 56–58, 27, 60] and not cost-effective in six. All studies finding panitumumab to be cost-effective were conducted in a pre-defined genetic group and therefore, further research is required comparing an alternative strategy where all patients receive standard of care without testing rather than that patients in comparator arm are all provided of panitumumab without biomarker testing. For example, two studies reported panitumumab as not cost-effective when compared with treating all patients with best supportive care without prior testing [61, 62].

Bevacizumab was evaluated only in three studies [66, 67, 58], two found it to be cost-effective and one not cost-effective. All three studies were in pre-defined patient groups. Ewara et al. [66] assessed first-line treatment strategies for mCRC patients with KRAS wild-type and compared three strategies of bevacizumab, cetuximab, panitumumab respectively combined with FOLFIRI and found that bevacizumab is dominant over both cetuximab and panitumumab. Similarly, Samyshkin et al. [58] also assessed three strategies of cetuximab, bevacizumab, and panitumumab for the first-line treatments for mCRC patients with KRAS wild-type, however, they found cetuximab plus FOLFIRI is the most cost-effective. But bevacizumab and panitumumab-containing regimens were also within the acceptable thresholds in UK. On the other hand, Lawrence et al. [67] found bevacizumab was not cost-effective with ICERs of $131,600 per QALYs, compared to fluoropyrimidine-based chemotherapy (FBC) alone.

## Quality assessment

The quality of the included studies was assessed by the Quality of Health Economic Studies (QHES) instrument. The QHES scale consists of 16 weighted questions, with a range of scores from 0 (worst quality) to 100 (best quality). The QHES tool was used by two independent assessors to rate the quality of the studies. QHES score per study is provided in S6 Table. Economic evaluations reported in full articles were scored using the QHES instrument (n = 30) and studies reported only in abstract (n = 16) were excluded from quality assessment due to their limited information.

In total, 60% of the studies scored above 70 (good quality) and 33% scored between 50 and 70 (fair quality), and only two papers scored below 50 (low quality). These scores were generated based on 16 ‘yes or no’ questions. The quality elements most commonly omitted from economic evaluations of cancer biomarkers for targeted therapies were the direction and magnitude of potential biases, the methodology for data abstraction, reliable use or justifications of health outcomes measures and scales. For the question “Did the author(s) explicitly discuss direction and magnitude of potential biases?” (Question 14), only 13% of articles were positively rated. With regard to health outcome measures (Question 11), only eight studies got positive scores. As for the question, “Was the methodology of data abstraction (including the value of health states and other benefits) stated?” (Question 7) 43% of articles were scored positively.

The study objectives were clearly presented by all studies (Question 1). The perspective of the analysis was not stated by Behl et al. [41] (Question 2). However, it seems plausible that Behl et al. might have used the perspective of US payer since, they briefly discussed the potential cost savings for the payer, chose the mCRC interventions most commonly used in USA and the analysis was commissioned by US National Institutes of Health. We found eleven papers [48, 37, 64, 45, 30, 73, 31, 65, 32, 36, 40] unlikely to have used data from best available source (Question 3). We interpreted this question as meaning that they provided insufficient justification of their choice of data sources. Applying data from another modelling paper or simply using RCT trial data without justifications (i.e. systematic literature review or meta-analysis) was considered insufficient. If estimates came from a subgroup analysis, were the groups pre-specified (Question 4). This item was not applicable for most of the studies since their estimates were not from a subgroup analysis. As for Question 5 on handling uncertainty, we awarded ‘yes’ to studies which performed at least one type of sensitivity analyses. We found that all studies performed one sensitivity analysis or more. However, five studies [30, 31, 35, 36, 68] only performed one-way sensitivity analysis which may be considered insufficient, for example, the NICE HTA guideline requires probabilistic sensitivity analysis [74]. Two studies did not perform incremental analysis between alternatives (Question 6) [35, 32]. Many studies did not clearly state the methodology for data abstraction of the values of health states and other benefits (n = 17) (Question 7). Four studies did not state the time horizon and discount rates applied in their studies (Question 8) [48, 63, 64, 45]. However, some studies justified that they did not discount their costs and benefits because of short time horizon of trial periods (18–19 months or 2 years) [72, 59], however this is not sufficient reason for not discounting and, to be appropriate methodologically, all costs and benefits beyond 1 year need to be discounted. Eight studies [66, 37, 30, 65, 35, 32, 40, 43] did not measure costs appropriately and the methodology for cost estimation was not clearly described (Question 9). Seven studies [49, 41, 63, 64, 59, 32, 36] did not clearly state primary outcome measures or did not provide clear descriptions of how they were measured (Question 10). Only eight studies [33, 51, 52, 62, 72, 29, 57, 65] used valid health outcomes and provided sufficient justifications for the measures and scales used (Question 11). Most other studies did not provide sufficient information on the health utility measures used or simply borrowed utility values from previous literature without justifications on validity of their measures and scales. Meanwhile, another eight studies did not include health outcomes at all and they estimated ICERs per LYs [48, 49, 41, 68, 64, 45, 30, 35]. Four studies were not transparent on their model structure and study methods including how they estimated monetary outcomes of cost-effectiveness (Question 12) [63, 72, 45, 65]. For example, Davari et al.[63] provided almost no information about their study methods and modelling structure. Most studies stated the choice of model and assumptions (n = 22) (Question 13). However, only four studies discussed potential biases in relation to their study results [33, 66, 62, 64] (Question 14). We found three studies did not come to a reasonable conclusion based on their study results (Question 15) but the conclusions of all other studies appear to be reasonable following their study results. However, three papers implied or suggested the intervention was cost-effective, while it was not cost-effective given the cost-effectiveness thresholds of the respective countries [48, 67, 32]. For example, Wen et al. calculated monthly estimations and thus, it should conclude that it is not cost-effective given the yearly WTP in China. All but three studies explicitly disclosed their funding source [48, 29, 31] (Question 16), although the Health Quality Ontario report is likely to be commissioned by public resources [29].

Finally, we also examined if there is any influence of commercial sponsorships in terms of the quality of economic evaluations and found that there is no influence. Among all eighteen studies rated as good quality (> = 70), ten studies were in fact funded by commercial sources mainly from manufacturers. However, all studies performed by public sources such as HTA bodies, i.e. NICE or Ontario HTA were very highly rated, above 85 scores [33, 61, 62, 29]. Overall, we found that most of the studies were of good or fair quality except for two papers which scored below 50.

## Discussion

Altogether, 46 papers were included in this systematic review. We identified three systematic reviews previously conducted for targeted therapies in mCRC [75–77], although they are different from ours in terms of the interventions focused. We focused on predictive biomarkers (or, companion biomarkers) and thus, targeted therapies with no licensed companion diagnostics were not included.

Our review is more comprehensive than previous studies. We identified and screened a much higher number of papers (n = 2893) and conducted longer periods of literature search (17.5 years between 2000 and June 2018). And finally, we included the highest number of studies in the review (n = 46) despite the narrower focus on predictive biomarkers with targeted therapies, while excluding cost-effectiveness analyses of targeted therapies with no licenced companion biomarkers.

Lange et al. [78] which focused on assessing the cost-effectiveness of monoclonal antibodies rather than that of biomarkers, is not directly comparable to our review. However, they provisionally suggested that KRAS testing is cost-effective compared to no-testing. They found that treatment with bevacizumab, cetuximab, and panitumumab was generally not cost-effective. They assessed the quality of identified papers but did not synthesize the results even qualitatively. Frank and Mittendorf [79] focused on pharmacogenomic profiling prior to the administration of pharmaceuticals in mCRC. They observed that the application of predictive biomarkers prior to EGFR antibodies was cost-effective but the cost-effectiveness of biomarkers for irinotecan-based chemotherapy remained unclear. They provided qualitative synthesis on key drivers and areas of uncertainty in the included studies. First, they found that biomarker costs were a driver of cost-effectiveness. Second, the characteristics of biomarkers such as performance accuracy and time of testing influence cost-effectiveness. Third, limited availability of clinical data is a source of uncertainty, especially because the efficacy of biomarkers is determined by the effects of subsequent therapies. Both reviews [78, 79] suggested that the addition of KRAS testing prior to treatment could be more cost-effective than a no-testing strategy. The most recent systematic literature review was done by Guglielmo et. al [77], focusing on genetic tests of Lynch syndrome (LS) and KRAS mutation tests. But their search covers a very short period and search strategies were not performed step by step. Overall, none of the studies synthesized the cost-effectiveness results of predictive biomarkers for corresponding therapies even qualitatively, although they assessed the quality of identified studies. To the best of our knowledge, this is the first paper that analysed the cost-effectiveness of predictive biomarkers and corresponding therapies separately and analysed the interactions between them in terms of the influence of predictive biomarkers on the cost-effectiveness of subsequent therapies.

We found that most studies used a third-party payer perspective such as health care systems or national health insurances, often taking account of only direct costs in their evaluations. Three studies included both direct and indirect costs from a societal perspective [36, 69, 71]. Zhou et al. [36] stated that they evaluated from a perspective of Chinese health care system, however, we categorised their study as having a societal perspective since they considered indirect costs as well i.e., travel fees and absenteeism fees. Although a general view is that it is appropriate to include both direct and indirect costs in cost-effectiveness analyses [19], it is not commonly practised in performing economic evaluations for pharmaceutical products especially when aimed to get reimbursed. Consequently, few economic evaluations have taken a societal perspective (n = 3) as seen in S4 Table. Without the changes to the HTA guidelines for reimbursement in respective countries, this trend won’t be reversed. For example, Krol et al. [71] conducted their study from two perspectives, a HCS perspective for Belgium and societal perspective for Netherlands, following the respective country’s HTA guidelines.

When conducting a comparative analysis such as cost-effectiveness analyses, it is methodologically and ethically important to use the most appropriate alternative therapy as a comparator strategy. Standard of care (SOC) is the most widely accepted comparator in economic evaluations according to cost-effectiveness analysis guidelines in many countries. However, we found that a majority of economic evaluations of biomarker-guided therapies were performed in a pre-defined genetic group (n = 23) and by doing so, most studies failed to explicitly consider the values of predictive biomarkers in their comparative analyses.

Our finding that whether the use of biomarkers makes corresponding therapies more cost-effective is largely driven by the expected impact on health outcomes rather than on costs contrasts with that of Frank and Mittendorf [79]. This finding also highlights that the cost-effectiveness analyses of targeted therapies should consider the sensitivity and specificity of biomarker testing. Our review showed that only six studies included the clinical characteristics of the biomarker such as performance accuracy [38, 28, 33, 37, 44, 35]. A considerable number of studies did not include this in their evaluations. For example, low sensitivity may lead to not giving targeted therapies to KRAS WT patients, whereas low specificity may lead to treating patients unresponsive to the therapy. Then, some of these patients may experience poorer outcomes owing to adverse events, compared to the comparator strategy of receiving BSC. Or, false negative test results may lead to not treating the responsive patients, which causes an accumulated loss of health benefits compared to the strategy of having all patients treated with the intervention without biomarker testing. Biomarker prevalence (proportion of patients with a biomarker status) was often not considered in evaluations.

Some limitations need to be acknowledged with regard to the present review. Systematic reviews are transparent, rigorous and reproducible and thus, are widely used to identify existing literature in many fields including health economics. However, literature searches using an electronic database may be limited by the performance of database filtering algorithms and indexers. Therefore, our review was supplemented by hand-searches using snowballing methods and references from other reviews as well as conference abstracts. Our review relies on published evidence in the public domain and consequently is vulnerable to publication bias. Given that quantitative synthesis of the study results of economic evaluations is not possible owing to heterogeneity across different countries and clinical settings, we performed the data synthesis qualitatively in order to provide a comprehensive view on the cost-effectiveness of predictive biomarkers for targeted therapies. As a typical example, economic evaluations of low income countries such as Chinese studies are not comparable to that of high income countries in terms of willingness to pay thresholds and healthcare systems.

In conclusion, companion biomarkers for targeted therapies in mCRC were mostly found to be cost-effective; otherwise, they improved the cost-effectiveness of corresponding therapies by saving some costs. However, they did not necessarily make the corresponding targeted therapies cost-effective. Biomarker’s clinical and economic inputs captured in economic evaluations of targeted therapies were often limited to the cost of tests and these values were frequently omitted especially when the scope of comparative analysis was limited to a pre-defined genetic population. In addition, we observed that there is no consensus on the best practice of strategy comparisons and no consistency in how to compare alternative strategies to estimate the ICERs of cancer biomarkers for targeted therapies in mCRC.

## Supporting information

S1 Table PRISMA checklist. (DOCX)

S2 Table Search terms. (DOCX)

S3 Table PICOS inclusion and exclusion criteria. (DOCX)

S4 Table Overview of included studies. (DOCX)

S5 Table Cost-effectiveness results of all included papers. (DOCX)

S6 Table QHES scoring per study. (DOCX)
