# Supplementary material for: Do cancer biomarkers make targeted therapies cost-effective? A systematic review in metastatic colorectal cancer
Source: PLoS One. 2018 Sep 26;13(9):e0204496. doi: 10.1371/journal.pone.0204496 (PMC6157891; doi:10.1371/journal.pone.0204496)
Supplement: S2 Table — (DOCX) [file pone.0204496.s002.docx]

**S2 Table. Search terms** (searched June 25, 2018)

*Database: EMBASE via Ovid*

| 1 | exp biological marker/ |
| --- | --- |
| 2 | biomark*.mp. [mp=title, abstract, heading word, drug trade name, original title, device manufacturer, drug manufacturer, device trade name, keyword, floating subheading] |
| 3 | (molecul* mark* or tumo?r mark* or biologic* mark* or signature molecule*).mp. [mp=title, abstract, heading word, drug trade name, original title, device manufacturer, drug manufacturer, device trade name, keyword, floating subheading] |
| 4 | (cetuximab or Erbitux* or panitumumab or Vectibix* or bevacizumab or avastin* or aflibercept or ziv-aflibercept or zaltrap* or regorafenib or stivarga* or ramucirumab or cyramza* or irinotecan or campto*).mp. [mp=title, abstract, heading word, drug trade name, original title, device manufacturer, drug manufacturer, device trade name, keyword, floating subheading] |
| 5 | (target therap* or targeted therap* or personali#ed medicine* or companion diagnostic* or precision medicine* or codependent technolog*).mp. [mp=title, abstract, heading word, drug trade name, original title, device manufacturer, drug manufacturer, device trade name, keyword, floating subheading] |
| 6 | exp economic evaluation/ |
| 7 | ((cost* adj3 effective*) or (cost* adj3 benefit*) or (cost* adj3 utilit*) or willingness to pay or net benefit*).mp. [mp=title, abstract, heading word, drug trade name, original title, device manufacturer, drug manufacturer, device trade name, keyword, floating subheading] |
| 8 | (econom* adj3 evaluation*).mp. [mp=title, abstract, heading word, drug trade name, original title, device manufacturer, drug manufacturer, device trade name, keyword, floating subheading] |
| 9 | exp colon tumor/ |
| 10 | 1 or 2 or 3 or 4 or 5 |
| 11 | 6 or 7 or 8 |
| 12 | 9 and 10 and 11 |

*Database: MEDLINE via Ovid*

| 1 | exp Biomarkers/ |
| --- | --- |
| 2 | biomark* |
| 3 | molecul* mark* OR tumo?r* mark* OR biologic* mark* OR signature molecule* |
| 4 | exp Clinical Laboratory Techniques/ |
| 5 | diagnos* |
| 6 | cetuximab or Erbitux* or panitumumab or Vectibix* or bevacizumab or avastin* or aflibercept or ziv-aflibercept or zaltrap* or regorafenib or stivarga* or ramucirumab or cyramza* or irinotecan or campto* |
| 7 | target therap* or targeted therap* or personali#ed medicine* or companion diagnostic* or precision medicine* or codependent technolog* |
| 8 | 1 or 2 or 3 or 4 or 5 or 6 or 7 |
| 9 | exp Cost-Benefit Analysis/ |
| 10 | econom* adj3 evaluation* |
| 11 | (cost* adj3 effective*) or (cost* adj3 benefit*) or (cost* adj3 utilit*) or willingness to pay or net benefit* |
| 12 | 9 or 10 or 11 |
| 13 | exp Colorectal Neoplasms/ |
| 14 | (colorectal or colon or colonic or bowel or rectum or rectal or intestin*) and (cancer* or tumo?r* or neoplasm* or carcinoma*) |
| 15 | 13 or 14 |
| 16 | 8 and 12 and 15 |

*Database: EconLit via Ovid*

| 1 | (biomark* or molecu* mark* or tumo?r mark* or biologic* mark* or signature molecule*).mp. [mp=heading words, abstract, title, country as subject] |
| --- | --- |
| 2 | (target therap* or targeted therap* or personali#ed medicine* or companion diagnostic* or precision medicine* or codependent technolog*).mp. [mp=heading words, abstract, title, country as subject] |
| 3 | ((colorectal or colon or colonic or bowel or rectum or rectal or intestin*) and (cancer* or tumo?r* or neoplasm* or carcinoma*)).mp. [mp=heading words, abstract, title, country as subject] |
| 4 | 1 or 2 or 3 |

*Databse : NHSEED*

| 1 | (biomarker*) |
| --- | --- |
| 2 | MeSH DESCRIPTOR Biomarkers EXPLODE ALL TREES IN NHSEED |
| 3 | MeSH DESCRIPTOR Clinical Laboratory Techniques EXPLODE ALL TREES IN NHSEED |
| 4 | (diagnos*) IN NHSEED |
| 5 | #1 OR #2 OR #3 OR #4 |
| 6 | MeSH DESCRIPTOR Colorectal Neoplasms EXPLODE ALL TREES IN NHSEED |
| 7 | #5 AND #6 |
