# Supplementary material for: Do cancer biomarkers make targeted therapies cost-effective? A systematic review in metastatic colorectal cancer
Source: PLoS One. 2018 Sep 26;13(9):e0204496. doi: 10.1371/journal.pone.0204496 (PMC6157891; doi:10.1371/journal.pone.0204496)
Supplement: S3 Table — (DOCX) [file pone.0204496.s003.docx]

**S3 Table. PICOS inclusion and exclusion criteria**

| **PICOS** | **Inclusion Criteria** | **Exclusion criteria** |
| --- | --- | --- |
| Population | Adult patients (>= 16 years) treated with metastatic CRC | Patients < 16 years Diagnosed mild CRC No diagnosed CRC |
| Intervention | Cancer biomarkers for targeted therapies. Companion biomarkers licensed with corresponding targeted therapies | No diagnostic biomarker  Universal screening tools Triage procedures Severity or progression analyses |
| Comparators | Targeted therapies with or without biomarkers | No comparative treatment Surgery |
| Outcomes | ICER, ICUR | Only costs or effectiveness |
| Study types | Economic evaluations (model or trial based CEA, CUA, CBA) | Cost-minimisation analysis No economic evaluations. Published only as an abstract without reporting any outcomes. Publications reporting merely on methodological issues, reviews, comment letters and editorials. Abstracts reported elsewhere - this criterion should only be applied if the numerical values are the same in the full publication.  NO English full-text. |
