# Supplementary material for: Do cancer biomarkers make targeted therapies cost-effective? A systematic review in metastatic colorectal cancer
Source: PLoS One. 2018 Sep 26;13(9):e0204496. doi: 10.1371/journal.pone.0204496 (PMC6157891; doi:10.1371/journal.pone.0204496)
Supplement: S4 Table — (DOCX) [file pone.0204496.s004.docx]

**S4 Table. Overview of included studies**

| **Study** | **Country** | **Perspective** | **Time horizon** | **Type of modeling** | **No. of strategies** | **Discount rate** | **Currency, price year** | **Funding** | **AB or FT** |
| --- | --- | --- | --- | --- | --- | --- | --- | --- | --- |
| Annemans et al. 2007 | Belgium | HCS | not reported | Trial-based model | 3 strategies | NR | Euro, NR | NR | FT |
| Asseburg et al. 2011 | Germany | HCS | 10-year | Patient-level simulation | 2 strategies | 5% | Euro, 2010 | Commercial resource | FT |
| Behl et al. 2012 | USA | Payer | 10 years | Markov model | 4 strategies | 3% | US$, 2010 | Public resource | FT |
| Blank et al. 2011 | Switzerzland | HCS | Lifetime | Markov model | 4 strategies | 3% | Euro, NR | Academic resource | FT |
| Butzke 2016 | Germany | HCS | Lifetime | Markov model | 3 strategies | 3% | Euro, 2013 | Public resource | FT |
| Carlson J.J. 2010 | USA | Payer perspective | NR | Unclear | 3 strategies | NR | US$, NR | NR | AB |
| Carvalho et al. 2017 | Brazil | Public healthcare system | Lifetime | Markov model | 3 strategies | 5% | US$, 2016 | Commercial resource | FT |
| Chaugule et al. 2012 | USA | Societal perspective | Lifetime | Markov model | 2 strategies | 3% | US$, NR | NR | AB |
| Davari et al. 2015 | Iran | Iranian health care market | Unclear/Not reported | Unclear | 6 strategies | NR | US$, NR | NR | FT |
| Dos Santos et al. 2015 | Brazil | Brazilian private healthcare system | Lifetime | Markov model | 2 strategies | 5% | Brazil local currency, 2014 | NR | AB |
| Ewara et al. 2014 | Canada Ontario | HCS | Lifetime | Markov model | 3 strategies | 5% | CA$, 2012 | Academic resource | FT |
| Gold et al. 2009 | USA | Medicare perspective | 5-year | Decision tree | 2 strategies | 3% | US$, 2007 | Academic resource | FT |
| Graham et al. 2014 | France | French health collective perspective | Lifetime | semi-Markov model | 2 strategies | 4% | Euro, 2013 | Commercial resource | FT |
| Graham et al. 2016 | USA | Third party payer | Lifetime | semi-Markov model | 2 strategies | 3% | US$, 2014 | Commercial resource | FT |
| Harty et al. 2018 | UK | UK NHS | 10-year | Markov model | 3 strategies | 3.5% | GB£, NR | Commercial resource | FT |
| Hnoosh et al. 2015 (AWMSG) | Wales, UK | NHS Wales | 10-year horizon | Markov model | 6 strategies | 3.5% | GB£, NR | NR | AB |
| Hnoosh et al. 2015 (NICE) | UK | NHS | 10-year | Markov model | 4 strategies | 3.5% | GB£, NR | NR | AB |
| Hoyle et al. 2013 | UK NICE | HCS | 10 years (lifetime) | Semi-Markov model | 4 strategies | 3.5% | GBP, 2011 | Public resource | FT |
| Huxley et al. 2017 | UK | NHS NICE | 30 years (lifetime) | Semi-Markov model | 3 strategies | 3.5% | GBP, 2015/16 | Public resource | FT |
| Junqueira et al. 2015 (subgroup, Cmab) | Brazil | Public healthcare system | 10 years | Markov model | 2 strategies | 5% | BRL, 2014 | NR | AB |
| Junqueira et al. 2015 (Cmab and Bmab) | Brazil | Public healthcare system | 10 years | Markov model | 2 strategies | 5% | BRL, 2014 | NR | AB |
| Kourlaba et al. 2014 | Greece | HCS | NR | Markov model | 2 strategies | NR | Euro, 2014 | NR | AB |
| Krol et al. 2015 | Netherlands, Belgium | Societal perspective (NL), Healthcare perpsective (BL) | 20-year horison | Markov model | 4 strategies | 4% (NL), 3%(BL) for costs and 1.5% for effect | Euro, NR | NR | AB |
| Lawrence et al. 2013 | Canada | Healthcare system | Lifetime (to maximum of 10 years) | Markov model | 4 strategies | 5% | CA$, 2011 | Commercial resource | FT |
| Mittmann 2009 | Canada | HCS | Duration of the clinical trial (18-19 months) | Trial-based model | 2 strategies | Discounting not applied given the short time horison | CA$, 2007 | No external funding | FT |
| Moreno et al. 2012 | Spain | not reported | not reported | Unclear | 3 strategies | not reported | Euro, NR | NR | AB |
| Niedersuess-Beke D. et al. 2015 | Austria | HCS | Not reported | Unclear | 2 strategies | NR | Euro, 2013 | NR | AB |
| Norum J. 2006 | Norway | third party payer | Unclear | Decision tree | 2 strategies | not discounted because all benefits and costs occurred within a few months. | NOK, 2005 | Public resource | FT |
| Obradovic et al. 2008 | USA | Healthcare payer | Lifetime | Decision tree | 3 strategies | not reported (looks more like discounting not applied) | US$, 2006 | Academic resource | FT |
| Ontario HTA 2010 | Ontario, Canada | HCS | Lifetime | Markov model | 7 strategies | 5% | CA$, 2009 | Public resource | FT |
| Ortendahl et al. 2014 | USA | Not reported | Lifetime | Unclear | 2 strategies | Not reported | US$, 2013 | NR | AB |
| Pichereau et al. 2010 | France | Hospital | Lifetime | Decision tree | 2 strategies | not reported | Euro, 2006 | No financial support | FT |
| Riesco-Martinez 2016 | Canada | Canadian public health care system | 5-year | Markov model | 3 strategies | 5% | CA$, 2012 | Commercial resource | FT |
| Rivera et al. 2017 | Spain | National Health System | Lifetime (max 20 years assumed) | Semi-Markov model | 2 strategies | 3% | Euro, 2015 | Commercial resource | FT |
| Saito et al. 2017 | Japan | Japanese healthcare payer | 5-year | Markov model | 3 strategies | 2% | JPY, NR | NR | FT |
| Samyshkin et al. 2011 | UK | UK NHS | Lifetime | semi-Markov model | 3 strategies | not reported | GBP, NR | NR | AB |
| Shankaran et al. 2015 | USA | Payer perspective | 2 years (trial period) | Decision tree | 2 strategies | 0% | US$, 2013 | Commercial resource | FT |
| Shiroiwa et al. 2010 | Japan | Healthcare payer | 2.5 years | Markov model | 3 strateges | 3% | US$, 2010 | Commercial resource | FT |
| Souza et al. 2017 | Brazil | Public health system perspective | 20 years | Markov model | 2 strategies | 5% | BRL, NR | Commercial resource | AB |
| Starling et al. 2007 | UK | NHS perspective | Lifetime | Trial-based model | 2 strategies | 3.5% | GB£, NR | Commercial resource | FT |
| Vargas-Valencia et al. 2015 | Columbia | Not reported | Lifetime | Markov model | 2 strategies | 5% | US$, NR | Not reported | AB |
| Vijayaraghavan et al. 2012 | USA, Germany | HCS | Lifetime | Markov model | 6 strategies | Not reported | US$, 2009  Euro, 2009 | Commercial resources | FT |
| Wen et al. 2015 | China | HCS | 10 years (almost lifetime; all nearly dead) | Markov model | 4 strategies | 3% | US$, 2014 | No funding | FT |
| Wu et al. 2017 | China | Chinese medical insurance perspective | 10 years | Markov model | 3 strategies | 5% | US$, 2016 | Public resource | FT |
| Xu et al. 2016 | USA | HCS (Medicare, Veteran) | 3 years | Markov model | 2 strategies | 3% | US$, 2015 | NR | AB |
| Zhou et al. 2016 | China | Societal perspective (because travel fees and absenteeism fees constituted the indirect costs). However the paper stated that it used Chinese HCS perspective | Lifetime | Markov model | Two comparative analyses were done.  Analysis 1 : 4 strategies  Analysis 2: 4 strategies | 3% | US$, NR | No funding | FT |

AB; abstract, FT; full text, NR; not reported,
