# Supplementary material for: Do cancer biomarkers make targeted therapies cost-effective? A systematic review in metastatic colorectal cancer
Source: PLoS One. 2018 Sep 26;13(9):e0204496. doi: 10.1371/journal.pone.0204496 (PMC6157891; doi:10.1371/journal.pone.0204496)
Supplement: S5 Table — (DOCX) [file pone.0204496.s005.docx]

**S5 Table. Cost-effectiveness results of all included papers**

| **Study**  **(reference)** | **Treatments/Strategies** | **Biomarker** | **Outcome measure** | **ICER (/LYs)** | **ICER (/QALYs)** | **Conclusion based on outcome** |
| --- | --- | --- | --- | --- | --- | --- |
| Annemans et al. 2007 | 1. Cmab + Irinotecan (6 week rule, 12 week rule)  2. Current treatment | NS | LYs | 1. Cmab + Irinotecan (6-week rule) : £16766  2. Cmab + Irinotecan (12-week rule) : £40273 | - | Cmab + Irinotecan is cost-effective in Belgium |
| Asseburg et al. 2011 | 1. Cmab + FOLIFIRI  2. Bmab + FOLFOX | KRAS | LYs | Cmab+FOLFIRI :€15,020 compared to Bmab + FOLFOX | - | First line treatment with Cmab plus FOLFIRI offers a cost-effective treatment option versus Bmab plus FOLFOX for KRAS WT genotype pts in Germany. Thus, KRAS testing should be performed on all presenting cases of mCRC to ensure access to this treatment option. |
| Behl et al. 2012 | 1. No Cmab  2. KRAS and BRAF testing + Cmab  3. KRAS testing + Cmab  4. Cmab without testing | KRAS,  BRAF | LYs | KRAS and BRAF testing + Cmab : US$648,396*  KRAS testing + Cmab : US$672,216*  Cmab without testing : US$827,913* | - | Screening for KRAS and BRAF improves the cost-effectiveness of Cmab. However, ICERs remain above the generally accepted threshold. Although we cannot confirm that Cmab is a cost-effective use of healthcare resources, we can confirm that KRAS testing is cost-saving. |
| Blank et al. 2011 | 1. No test, no Cmab  2. KRAS/BRAF testing  3. KRAS testing  4. No test, Cmab all | KRAS,  BRAF | QALYs | 1. no test, no Cmab (reference strategy) : dominated  2. KRAS/BRAF testing : euro 62,653 compared to the reference strategy  3. KRAS testing : euro 313,537 compared KRAS/BRAF testing  4. No test, Cmab all : euro 314,588 compared to KRAS testing | - | Testing for KRAS and BRAF mutations prior to Cmab treatment of chemofractory mCRC patients is clinically appropriate and economically favorable, despite high costs for predictive testing. |
| Butzke 2016 | Strategy 1. dose reduction (WT receive standard dose of irinotecan, hetero-and homozygotes receive a dose reduction of irinotecan by 25%)  Strategy 2. prophylactic administration of bon marrow proective GCSF growth factor analogs (all pts receive standard dose of irinotecan, hetero-and homozygotes additionally receive the growth factor 'pegfilgrastim')  Strategy 3. no genetic test (all pts receive standard dose of irinotecan) | UGT1A1 | QALYs | - | Genetic test + irinotecan (dose reduction): dominant over two other strategies. | UGT1A1 testing and dose reductionn is more effective and cost-saving compared to the current standard of no-testing. UGT1A1 testing prior to irinotecan-based chemotherapy dominates non-personalised care in Germany. |
| Carlson J.J. 2010  (abstract) | 1. Cmab alone  2. BSC  3. KRAS testing plus Cmab for KRAS WT pts and BSC for KRAS MT pts | KRAS | QALYs | Cmab for all : $357,224 compared to BSC  KRAS testing + Cmab : $264,644 | - | Use of KRAS testing to select pts for Cmab can reduce costs with a negligible impact on QALYs as compared to using Cmab for all pts. However, the CE of KRAS testing vs. BSC remains well above commonly used cost-effectiveness thresholds |
| Carvalho et al. 2017 | 1. Pmab 2. Cmab 3. BSC | RAS | LYs | 1. Pmab: US$52772 2. Cmab: US$58240 | - | Both Pmab and Cmab are not cost-effective in patients with RAS WT mCRC |
| Chaugule et al. 2012 | 1. Cmab + BSC  2. BSC alone | KRAS | QALYs | - | Cmab + BSC: US$ 313,113 compared to BSC | Cmab is not cost-effective in KRAS WT pts with mCRC |
| Davari et al. 2015 | 1. FOLFIRI, FOLFOX, CAPOX without the addition of Cmab  2. FOLFIRI, FOLFOX, CAPOX with the addition of Cmab | KRAS | LYs, QALYs | 1. FOLFIRI vs. FOLFIRI+Cmab $654846  2. FOLFOX vs. FOLFOX+Cmab $458113  3. CAPOX vs. CAPOX+Cmab $461989 | 1. FOLFIRI vs. FOLFIRI+Cmab $859756  2. FOLFOX vs. FOLFOX+Cmab $1588143  3. CAPOX vs. CAPOX+Cmab $1567786 | Addition of Cmab to FOLFIRI, FOLFOX, CAPOX (Capecitabin+oxaliplati) is not cost effective |
| Dos Santos et al. 2015  (Abstract) | 1. Pmab + mFOLFOX6  2. Bmab + mFOLFOX6 | RAS | LYs, QALYs | 1. Pmab + mFOLFOX6 vs. Bmab + mFOLFOX6 : 25,798 BRL per LYs gained | 1. Pmab + mFOLFOX6 vs. Bmab + mFOLFOX6 : 34,960 BRL per QALYs gained | Pmab is clearly cost-effective compared to Bmab for treatment of wild-type RAS mCRC in Brazil. |
| Ewara et al. 2014 | 1. Bmab + FOLFIRI  2. Cmab + FOLFIRI  3. Pmab + FOLFIRI | KRAS | QALYs | - | 1. Bmab + FOLFIRI : Dominant  2. Cmab + FOLFIRI : Dominated  3. Pmab + FOLFIRI : Dominated | Bmab+FOLFIRI is cost-effective. Bmab + FOLFIRI found to be dominant over the other two strategies. The other two strategies are dominated by Bmab + FOLFIRI. However, sensivitiy analysis showed that Cmab + FOLIFIRI is being cost-effective under certain range of parameter values - thus, further investigation needed for Cmab. |
| Gold et al. 2009 | 1. Usual care : all pts receive a standard intermediate dose of irinotecan.  2. Genetic testing strategy : test + Irinotecan | UGT1A1 | QALYs | - | Genetic testing + Irinotecan : Dominant compard to no testing strategy | Pharmaceogenetic testing for UGT1A1*28 variant homozygosity may be cost-effective, but only if irinotecan dose reduction in homozygotes does not reduce efficacy. Future studies to evaluate reduced-dose efficacy in homozygotes should be considered. |
| Graham et al. 2014 | 1. Pmab  2. Bmab | KRAS, RAS | LYs, QALYs | Pmab : €26,918 | Pmab : €36,577 | Pmab plus mFOLFOX represents good value for money compared to a current SOC Bmab plus mFOLFOX6 |
| Graham et al. 2016 | 1. Panitumumab in pts with KRAS WT status  2. Cetuximab in pts with KRAS WT status | KRAS | LYs, QALYs | Dominant (panitumumab dominates) -$307,432 | Dominant (panitumumab dominates) -$648,345 | Compared to Cmab, the study suggested that Pmab is favorable. |
| Harty et al. 2018 | 1. Cmab+FOLFIRI  2. FOLFIRI | KRAS, RAS | QALYs | - | 3 cohorts compared  ITT (intention-to-treat) group: £130,929  KRAS WT group: £72,053  RAS WT group: £44,185 | RAS WT group showed the lowest ICER and thus, it is the most cost-effective of the three groups |
| Hnoosh et al. 2015 (AWMSG)  (Abstract) | 1. Cmab + either FOLFOX, FOLFIRI, CAPOX  2. FOLFOX  3. FOLFIRI  4. CAPOX | RAS | QALYs | - | Cmab + FOLFOX £29,512 compared to FOLFOX alone.  Cmab + FOLFIRI £35,731 compared to FOLFIRI alone. | Cmab is cost-effective and a good use of NHS Wales resource through stratifiation of RAS wild-type patients |
| Hnoosh et al. 2015 (NICE)  (Abstract) | 1. Cmab + either FOLFOX, FOLFIRI, CAPOX  2. FOLFOX  3. FOLFIRI | RAS | QALYs | - | Cmab+FOLFOX: £46503 compared to FOLFOX alone  Cmab+FOLFIRI: £55971 compared to FOLFIRI alone | Cost-effectiveness of Cmab could be deemed favourable when considering it as end-of-life medicine |
| Hoyle et al. 2013 | 1. Cmab  2. Cmab + Irinotecan  3. Pmab  4. BSC | KRAS | LYs, QALYs | 1. Cmab £72,000 compared to BSC  2. Cmab + Irinotecan £64,000 compared to BSC  3. Pmab £153,000 compared to BSC | 1. Cmab £95,000 compared to BSC  2. Cmab + Irinotecan £88,000 compared to BSC  3. Pmab £187,000 compared to BSC | All three strategies (Cmab, Cmab+Irinotecan, Pmab) are not cost-effective. |
| Huxley et al. 2017 | 1. FOLFOX (reference strategy)  2. Cmab + FOLFOX  3. Pmab + FOLFOX | RAS | QALYs | - | Cmab+FOLFOX vs. FOLFOX : £104205 per QALYs gained  Pmab + FOLFOX vs. FOLFOX : £204103 per QALY gained | Cmab and Pmab in combination with chemotherapy are likely to be poor value for money |
| Junqueira et al. 2015 (RAS subgroup)  (Abstract) | 1. Cmab + FOLIFIRI  2. FOLFIRI | RAS | LYs | BRL 66090.91 | - | Cmab+FOLIFIRI is cost-effective for a subgroup of patients with RAS wild-type |
| Junqueira et al. 2015 (Cmab and Bmab)  (Abstract) | 1.Cmab+FOLFIRI  2.Bmab+FOLFIRI | RAS | LYs | Cmab+FOLFIRI: dominant, cost-saving | - | The use of Cmab shown significant and meaningful benefits while being cost-saving to HCS in Brazil. |
| Kourlaba et al. 2014  (Abstract) | 1. Pmab + FOLFOX6  2. Bmab + FOLFOX6 | RAS | QALYs | - | 1. Pmab + FOLFOX6 : €34,644 compared to Bmab + FOLFOX6 | Pmab + mFOLFOX6 is cost-effective. |
| Krol et al. 2015  (Abstract) | 1. Cmab + FOLFIRI  2. FOLFIRI  3. Cmab + FOLFOX  4. FOLFOX | RAS | QALYs | - | 1. 86180euro (NL) and €55430(BL) for Cmab + FOLFIRI vs. FOLFIRI | ICUR results were close to CET. ICURs strongly differed from NL and BL. It is mainly due to lower drug costs in BL. |
| Lawrence et al. 2013 | 1. FBC  2. Bmab + FBC  3. Cmab + FBC  4. Pmab + FBC | KRAS | QALYs | - | FBC -  Bmab + FBC : CA$131,600  Pmab + FBC : Dominated  Cmab + FBC : CA$3,844,571 | Bmab + FBC offers the best value for money in KRAS wt patient population. |
| Mittmann 2009 | 1. Cmab + BSC  2. BSC | KRAS | LYs, QALYs | 1. For unselected mCRC pts, Cmab+BSC: CA$199,742 compared to BSC.  2. For KRAS WT pts, Cmab + BSC: CA$120,061 compared to BSC. | 1. For unselected mCRC pts, Cmab + BSC : CA$299,613 compared to BSC.  2. For KRAS WT pts, Cmab+BSC: CA$186,761 compared to BSC. | ICER of Cmab over BSC alone for unselected mCRC pts was high and sensitive to drug costs. ICER was lower when the analysis was limited to pts with KRAS WT. |
| Moreno et al. 2012  (Abstract) | 1. Scenario A: KRAS WT pts receive weekly Cmab + FOLFOX  2. Scenario B. Pmab + FOLFOX  3. Scenario C. Cmab biweekly + FOLFOX | KRAS | Response rate | Scenario A vs. B : €4394  Scenario C vs. B : €4432 | - | 1st line oxaplatin combinations of biweekly Cmab for WT and Bmab for MT optimise cost per additional response rate rather than Pmab-based schedules |
| Niedersuess-Beke D. et al. 2015 | 1.Predictive biomarker testing  2.No predictive biomarker testing | KRAS, RAS, future biomarker | Lys | €26.276 (KRAS testing scenario)  €9.686 (RAS testing scenario)  €3.948 (future but achievable biomarker scenario) | - | Testing predictive biomarkers is cost-saving in mCRC |
| Norum J. 2006 | 1. 3rd line chemotherapy (Cmab + Irinotecan)  2. No 3rd line chemotherapy | EGFR | LYs | The range of ICERs was between €205,536 and €323,040 | - | Cmab + Irinotecan as 3rd line therapy in mCRC is promising, but a very expensive antibody. Reduced drug cost and/or improved overall survival may alter this conclusion |
| Obradovic et al. 2008 | 1. No UGT1A1 genotyping + SOC  2. UGT1A1 testing + Reduced initial ironotecan dose (20% reduction)  3. UGT1A1 testing + standard irinotecan dose+ Prophylactic use of GCSF | UGT1A1 | Severe neutropenia occurance (severe neutropenida prevention), LYs | 1. Genotyping + reduced initial irinotecan dose :  (African group) : cost-saving compared to No genotyping strategy  (Asian group) : US$6,818,203  (Caucasian group) : cost-saving compared to No genotyping strategy  2. Genotyping + Prophylatic use of GCSF :  (African group) : US$3,506,260  (Asian group) : US$7,371,770  (Caucasian group) : US$3,836,998 | - | Genotyping in combination with reduced irinotecan dose for genotype pts was cost-saving for the population of African and Caucasian origin. By contrast, genotyping was not cost-effective for the population of Asian ancestry. The prophylactic use of GCSFs in genotype pts was not cost-effective for any population group. |
| Ontario HTA 2010 | 0. BSC (no KRAS test; no treatment)  1a. KRAS testing + Cmab  1b. No KRAS testing + Cmab  2a. KRAS testing + Pmab  2b. No KRAS testing + Pmab  3a. KRAS testing + Cmab + Irinotecan  3b. No KRAS testing + Cmab + Irinotecan | KRAS | QALYs | - | 0. BSC (no KRAS test; no treatment)  1a. KRAS testing + Cmab : $54,802 compared to BSC  1b. No KRAS testing + Cmab : Dominated compared to BSC  0. BSC (no KRAS test; no treatment)  2a. KRAS testing + Pmab : $47,795 compared to BSC  2b. No KRAS testing + Pmab : $308,236 compared to BSC  0. BSC (no KRAS test; no treatment)  3a. KRAS testing + Cmab + Irinotecan : $42,710 compared to BSC  3b. No KRAS testing + Cmab + Irinotecan : $163,396 compared to BSC | All strategies considering KRAS testing found to be cost-effective compared to no-KRAS-testing strategies. |
| Ortendahl et al. 2014 | 1. FOLFIRI + Cmab  2. FOLFIRI + Bmab | KRAS, RAS | LYs, QALYs | 1. For KRAS WT pts, FOLIFIRI + Cmab : US$97297 compared to Bmab + FOLFIRI  2. For a subset of RAS WT pts, FOLFIRI + Cmab : US$77380 compared to Bmab + FOLFIRI | 1. For KRAS WT pts, FOLIFIRI + Cmab : US$122704 compared to Bmab + FOLFIRI  2. For a subset of RAS WT pts, FOLFIRI + Cmab : US$99,636 compared to Bmab + FOLFIRI | Cmab + FOLFIRI improve health outcomes and use financial resource more efficiently compared to Bmab + FOLFIRI |
| Pichereau et al. 2010 | 1. UGT1A1 genotyping + irinotecan therapy  2. No UGT1A1 genotyping + Irinotecan therapy | UGT1A1 | Number of neutropenia avoided | Genotyping strategy : €942.8 to €1090.1 | - | UGT1A1 genotype screening before irinotecan treatment is a cost-effective strategy for the hospital. |
| Riesco-Martinez 2016 | Strategy 1 (reference strategy: EGFRI monotherapy in 3rd line).  1st LINE : Bmab+FOLFIRI/FOLFOX (1st Line), 2nd LINE : FOLFIRI/FOLFOX, 3rd LINE : EGFRI  Strategy 2 (EGFRI and Irinotecan in 3L).  1L : Bmab+FOLFIRI/FOLFOX, 2L : FOLFIRI/FOLFOX, 3L : EGFRI + irinotecan  Strategy 3 (EGFRI in 1L).  1L : EGFRI + FOLFIRI/FOLFOX, 2L : Bmab + FOLFIRI/FOLFOX, 3L : best supportive care | KRAS, RAS | QALYs | - | Strategy 2 : $119,623 compared to Strategy 1  Strategy 3 : $3,176,591 compared to Strategy 1 | 1st line of EGFRI is not cost-effective at its current pricing relative to Bmab |
| Rivera et al. 2017 | 1. Pmab + mFOLFOX6  2. Bmab + mFOLFOX6 | RAS | LYs, QALYs | €16,567 | €22,794 | Pmab+mFOLFOX6 is more cost-effective than Bmab+mFOLFOX6 for the first line treatment of RAS wild-type mCRC |
| Saito et al. 2017 | No testing strategy: Anti-EGFR therapy without testing  2. RAS screening : RAS mutation screening before anti-EFGR therapy  3. Comprehensive screening : comprehensive molecular profiling before anti-EGFR therapy using CancerPlex to screen for mutations that predict a poor response. | RAS | LYs, QALYs | RAS screening: JYP2,574,111  Comprehensive screening: JYP3,587,395 | RAS screening: JYP3,049,132  Comprehensive screening: JYP4,260,187 | Comprehensive screening (comprehensive molecular profiling) is more cost-effective than RAS screening before administering anti-EGFR therapies |
| Samyshkin et al. 2011 | 1. Bmab + Chemotherapy  2. Cmab + Chemotherapy  3. Pmab + Chemotherapy | KRAS | QALYs | - | Cmab+FOLFIRI : £30,665 compared to FOLFIRI alone.  Bmab + FOLFOX : £17,626 compared to FOLFOX alone.  Pmab + FOLFOX : £15,326 compared to FOLFOX alone. | Cmab plus FOLFIRI is the most cost-effective for pts with KRAS WT tumors. ICERs of Cmab + Chemotherapy (CT), Bmab + CT, and Pmab + CT are within the commonly accepted threshold of CE in UK |
| Shankaran et al. 2015 | 1. FOLFIRI plus Cmab in treatment-naïve patients with KRAS wt type in mCRC  2. FOLFIRI plus Bmab treatment-naïve patients with KRAS wt type in mCRC | KRAS, RAS | LYs, QALYs | KRAS-WT patients:  Cmab+FOLIFIRI $86,487per LY  RAS-WT patients:  Cmab_FOLIFIRI $73,731 per LY | KRAS-WT patients:  Cmab+FOLIFIRI $107,630 per QALY  RAS-WT patients:  Cmab+FOLIFIRI $93,785 per QALY | Results were more favorable for Cmab in RAS-WT patients |
| Shiroiwa et al. 2010 | 1. KRAS testing + Cmab (WT - Cmab, MT - BSC) : Strategy A  2. No KRAS testing + all Cmab : Strategy B  3. No KRAS testing + all BSC : Strategy C | KRAS | LYs, QALYs | 1. KRAS testing + Cmab : dominant compared to no KRAS testing  2. KRAS testing : US$120,000 compared to no-Cmab strategy  3. No KRAS testing : US$160,000 compared to no Cmab strategy | 1. KRAS testing + Cmab : dominant compared to no KRAS testing  2. KRAS testing : US$180,000 compared to no-Cmab strategy  3. No KRAS testing : US$230,000 compared to no Cmab strategy | KRAS testing is dominant compared to no-KRAS testing strategy. However, ICER of Cmab + KRAS testing is US$180,000 per QALYs compared to no-Cmab strategy. |
| Souza et al. 2017 | 1. Cmab + Chemotherapy  2. Chemotherapy alone | RAS | LYs | R$56,750 | - | The addition of Cmab to the standard chemotherapy is cost-effective |
| Starling et al. 2007 | 1. Cmab + Irinotecan  2. Active/best supportive care (ASC/BSC) | EGFR | LYs, QALYs | £42,975 | £57,608 | ICERs for Cmab+Irinotecan is relatively high compared to other healthcare interventions. |
| Vargas-Valencia et al. 2015 | 1. Pmab + FOLFOX  2. Cmab + FOLFIRI | RAS | LYs | Pmab + FOLFOX US$ 21,613.42  Cmab + FOLFIRI US$ 23,036.94 | - | Pmab showed treatment outcomes improvement vs. Cmab for wt RAS pts at a lower cost per life year. |
| Vijayaraghavan et al. 2012 | 1. KRAS testing + Cmab  2. KRAS testing + Pmab  3. KRAS testing + Combination therapy (Cmab + Irinotecan)  4. No testing + Cmab  5. No testing + Pmab  6. No testing + Combination therapy (Cmab + Irinotecan) | KRAS | LYs | 1. No testing + Pmab : higher cost same effectiveness compared to KRAS testing + Pmab  2. No testing + Cmab : higher cost same effectiveness compared to KRAS testing + Cmab  3. No testing + Combination therapy : higher cost same effectiveness compared to KRAS testing + Combination therapy | - | Using KRAS testing to limit use of EGFR inhibitors (Cmab/Pmab) to pts with KRAS WT results in net savings of $7500 to $12400 (for USA), while net savings of €3900 to €9600 (for Germany) |
| Wen et al. 2015 | 1. Pts with KRAS testing treated with Cmab and FOLFIRI (KRAS-Cmab)  2. Pts with RAS testing treated with Cmab and FOLFIRI (RAS-Cmab)  3. Pts with KRAS testing treated with Bmab and FOLFIRI (KRAS-Bmab)  4. Pts with RAS testing treated with Bmab and FOLFIRI (RAS-Bmab) | KRAS, RAS | Quality-adjusted life-months (QALMs) |  | 1st and 2nd line  KRAS-Bmab $6,145.84 per QALMs  RAS-Bmab $6,201.34 per QALMs  RAS-Cmab $6,263.86 per QALMs  KRAS-Cmab $6,963.70 per QALMs | RAS screening prior to Cmab seems to be a cost-effective strateggy in the time of monoclonal antibodies therapies. However, KRAS-Cmab strategy dominated by other 3 strategies. RAS-Bmab seems the most cost-effective strategy but the gained QALM was the shortest. Compared to other 3 strategies, RAS-Cmab achieved the highest gained QALM of 21.85 |
| Wu et al. 2017 | 1. Cmab + FOLFIRI (with or without patient assistance programme)  2. FOLFIRI | RAS | LYs, QALYs | Cmab + FOLFIRI with PAP: $12,107  Cmab + FOLFIRI without PAP: $23,393 | Cmab + FOLFIRI with PAP: $14,049  Cmab + FOLFIRI without PAP: $27,145 | RAS testing with Cmab is cost-effective, when PAP is available, at a willingness to pay threshold of China ($22,200/QALY) |
| Xu et al. 2016 | 1.Pmab  2.Cmab | NR | LYs, QALYs | - | - | Pmab dominates over Cmab. Pmab has a cost advantage over Cmab |
| Zhou et al. 2016 | Analysis I:  1. KRAS-Cmab  2. KRAS-Bmab  3. RAS-Cmab  4. RAS-Bmab  Analysis II:  1. FOLFOX-Cmab  2. FOLFOX-Bmab  3. FOLFIRI-Cmab  4. FOLFOX-Bmab | KRAS, RAS | Quality-adjusted life years (QALYs) | - | Analysis I (KRAS-wt vs. RAS-wt):  1. KRAS-Cmab (Dominated) $88,394.09  2. KRAS-Bmab (Dominated) $80,797.82  3. RAS-Cmab (420,700.50 compared to RAS-Bmab) $82,590.72  4. RAS-Bmab (n.a) $75.358.42  Analysis II (RAS wt):  1. FOLFOX-Cmab (Dominated)  2. FOLFOX-Bmab (Dominated)  3. FOLFIRI-Cmab (Dominated)  4. FOLFOX-Bmab (Undominated) | Analysis 1 : RAS wt testing is more cost-effective than KRAS wt testing before treatment. It is the first head to head cost-effectiveness study to evaluate predictive testing for extended RAS-wt in mCRC in the context of targeting Cmab/Bmab treatment. The results demonstrates that it was economically favorable to identify pts with extended RAS-wt status. Furthermore, FOLFIRI plus Bmab was the preferrred strategy compared with other strategies in pts with extended RAS wt. |

*Recalculated compared to No Cmab strategy

Bmab, bevacizumab; Cmab, Cetuximab; EGFR, epidermal growth factor receptor; ICER, incremental cost-effectiveness ratio; KRAS, kirsten rat sarcoma; RAS, rat sarcoma; LYs, life years; NS, not specified; NR, not reported; PAP, patient assistance programme; Pmab, panitumumab; QALYs, quality adjusted life years; QALMs, quality adjusted life months; SOC, standard of care; WT, wild-type
