# Supplementary material for: Do cancer biomarkers make targeted therapies cost-effective? A systematic review in metastatic colorectal cancer
Source: PLoS One. 2018 Sep 26;13(9):e0204496. doi: 10.1371/journal.pone.0204496 (PMC6157891; doi:10.1371/journal.pone.0204496)
Supplement: S6 Table — (DOCX) [file pone.0204496.s006.docx]

**S6 Table. QHES scoring per study**

| Study | 1 | 2 | 3 | 4 | 5 | 6 | 7 | 8 | 9 | 10 | 11 | 12 | 13 | 14 | 15 | 16 | Score |
| --- | --- | --- | --- | --- | --- | --- | --- | --- | --- | --- | --- | --- | --- | --- | --- | --- | --- |
| Annemans et al. 2007 | yes | yes | no | no | yes | yes | no | no | yes | yes | no | yes | no | no | no | no | 48 |
| Asseburg et al. 2011 | yes | yes | yes | n/a | yes | yes | no | yes | yes | no | no | yes | yes | no | yes | yes | 75 |
| Behl et al. 2012 | yes | no | yes | n/a | yes | yes | yes | yes | yes | no | no | yes | yes | no | yes | yes | 76 |
| Blank et al. 2011 | yes | yes | yes | n/a | yes | yes | no | yes | yes | yes | no | yes | yes | no | yes | yes | 81 |
| Butzke 2016 | yes | yes | yes | n/a | yes | yes | yes | yes | yes | yes | yes | yes | yes | yes | yes | yes | 99 |
| Carvalho et al. 2017 | yes | yes | yes | no | yes | yes | no | yes | yes | yes | no | yes | yes | no | yes | yes | 81 |
| Davari et al. 2015 | yes | yes | yes | n/a | yes | yes | no | no | yes | no | no | no | no | no | yes | yes | 53 |
| Ewara et al. 2014 | yes | yes | yes | n/a | yes | yes | yes | yes | no | yes | no | yes | yes | yes | yes | yes | 84 |
| Gold et al. 2009 | yes | yes | no | n/a | yes | yes | no | yes | no | yes | no | yes | yes | no | yes | yes | 65 |
| Graham et al. 2014 | yes | yes | yes | n/a | yes | yes | no | yes | yes | yes | yes | yes | yes | no | yes | yes | 88 |
| Graham et al. 2016 | yes | yes | yes | n/a | yes | yes | yes | yes | yes | yes | yes | yes | yes | no | yes | yes | 93 |
| Harty et al. 2018 | yes | yes | yes | yes | yes | yes | yes | yes | no | yes | no | yes | no | no | yes | yes | 72 |
| Hoyle et al. 2013 | yes | yes | yes | n/a | yes | yes | yes | yes | yes | yes | no | yes | yes | no | yes | yes | 86 |
| Huxley et al. 2017 | yes | yes | yes | yes | yes | yes | yes | yes | yes | yes | yes | yes | yes | yes | yes | yes | 100 |
| Lawrence et al. 2013 | yes | yes | yes | n/a | yes | yes | no | yes | yes | yes | no | yes | yes | no | no | yes | 73 |
| Mittmann 2009 | yes | yes | yes | yes | yes | yes | yes | no | yes | yes | yes | no | yes | no | yes | yes | 79 |
| Norum J. 2006 | yes | yes | no | n/a | yes | yes | no | no | yes | no | no | yes | yes | yes | yes | yes | 66 |
| Obradovic et al. 2008 | yes | yes | no | yes | yes | yes | no | no | yes | yes | no | no | no | no | yes | yes | 52 |
| Ontario HTA 2010 | yes | yes | yes | yes | yes | yes | yes | yes | yes | yes | yes | yes | yes | no | yes | no | 91 |
| Pichereau et al. 2010 | yes | yes | no | n/a | yes | yes | no | yes | no | yes | no | yes | yes | no | yes | yes | 65 |
| Riesco-Martinez 2016 | yes | yes | no | n/a | yes | yes | yes | yes | yes | yes | no | yes | no | no | yes | yes | 71 |
| Rivera et al. 2017 | yes | yes | yes | no | yes | yes | yes | yes | yes | yes | yes | yes | yes | no | yes | yes | 93 |
| Saito et al. 2017 | yes | yes | no | no | yes | yes | no | yes | yes | yes | no | yes | yes | no | yes | no | 70 |
| Shankaran et al. 2015 | yes | yes | yes | n/a | yes | yes | no | no | yes | no | no | yes | yes | no | yes | yes | 68 |
| Shiroiwa et al. 2010 | yes | yes | yes | yes | yes | yes | yes | yes | yes | yes | no | yes | yes | no | yes | yes | 87 |
| Starling et al. 2007 | yes | yes | no | n/a | yes | yes | yes | yes | no | yes | yes | no | yes | no | yes | yes | 69 |
| Vijayaraghavan et al. 2012 | yes | yes | yes | n/a | yes | no | no | yes | no | yes | no | yes | yes | no | yes | yes | 67 |
| Wen et al. 2015 | yes | yes | no | n/a | yes | no | no | yes | no | no | no | yes | no | no | no | yes | 38 |
| Wu et al. 2017 | yes | yes | no | no | yes | yes | no | yes | no | yes | no | yes | no | no | yes | yes | 58 |
| Zhou et al. 2016 | yes | yes | no | n/a | yes | yes | no | yes | yes | no | no | yes | no | no | yes | yes | 60 |
